# Effect of long-term caloric restriction on DNA methylation measures of biological aging in healthy adults from the CALERIE trial

**DOI:** 10.1038/s43587-022-00357-y

**Published:** 2023-02-09

**Authors:** R. Waziry, C. P. Ryan, D. L. Corcoran, K. M. Huffman, M. S. Kobor, M. Kothari, G. H. Graf, V. B. Kraus, W. E. Kraus, D. T. S. Lin, C. F. Pieper, M. E. Ramaker, M. Bhapkar, S. K. Das, L. Ferrucci, W. J. Hastings, M. Kebbe, D. C. Parker, S. B. Racette, I. Shalev, B. Schilling, D. W. Belsky

**Affiliations:** 1 Butler Columbia Aging Center, Columbia University Mailman School of Public Health, New York, NY USA; 2 Department of Genetics, University of North Carolina at Chapel Hill School of Medicine, Chapel Hill, NC USA; 3 Duke Molecular Physiology Institute and Department of Medicine, Duke University School of Medicine, Durham, NC USA; 4 Department of Medical Genetics, Edwin S.H. Leong Healthy Aging Program, Centre for Molecular Medicine and Therapeutics, University of British Columbia, Vancouver, British Columbia Canada; 5 Department of Epidemiology, Columbia University Mailman School of Public Health, New York, NY USA; 6 Center on Aging and Development, Biostatistics and Bioinformatics, Duke University, Durham, NC USA; 7 Duke Clinical Research Institute, Duke University School of Medicine, Durham, NC USA; 8 Jean Mayer USDA Human Nutrition Research Center on Aging at Tufts University, Boston, MA USA; 9 Translational Gerontology Branch, National Institute on Aging, National Institutes of Health, Baltimore, MD USA; 10 Department of Biobehavioral Health, Pennsylvania State University, State College, PA USA; 11 Pennington Biomedical Research Center, Baton Rouge, LA USA; 12 Department of Medicine, Duke University School of Medicine, Durham, NC USA; 13 Program in Physical Therapy and Department of Medicine, Washington University School of Medicine, St. Louis, MO USA; 14 College of Health Solutions, Arizona State University, Phoenix, AZ USA; 15 Buck Institute for Research on Aging, Novato, CA USA

**Keywords:** Predictive markers, Predictive markers

## Abstract

The geroscience hypothesis proposes that therapy to slow or reverse molecular changes that occur with aging can delay or prevent multiple chronic diseases and extend healthy lifespan^1–3^. Caloric restriction (CR), defined as lessening caloric intake without depriving essential nutrients^4^, results in changes in molecular processes that have been associated with aging, including DNA methylation (DNAm)^5–7^, and is established to increase healthy lifespan in multiple species^8,9^. Here we report the results of a post hoc analysis of the influence of CR on DNAm measures of aging in blood samples from the Comprehensive Assessment of Long-term Effects of Reducing Intake of Energy (CALERIE) trial, a randomized controlled trial in which *n* = 220 adults without obesity were randomized to 25% CR or ad libitum control diet for 2 yr (ref. ^10^). We found that CALERIE intervention slowed the pace of aging, as measured by the DunedinPACE DNAm algorithm, but did not lead to significant changes in biological age estimates measured by various DNAm clocks including PhenoAge and GrimAge. Treatment effect sizes were small. Nevertheless, modest slowing of the pace of aging can have profound effects on population health^11–13^. The finding that CR modified DunedinPACE in a randomized controlled trial supports the geroscience hypothesis, building on evidence from small and uncontrolled studies^14–16^ and contrasting with reports that biological aging may not be modifiable^17^. Ultimately, a conclusive test of the geroscience hypothesis will require trials with long-term follow-up to establish effects of intervention on primary healthy-aging endpoints, including incidence of chronic disease and mortality^18–20^.

## Abstract (web summary)

Using samples and data from the CALERIE study, a human randomized controlled trial of long-term caloric restriction, the authors find that the intervention slows DunedinPACE, a DNA methylation measure of the pace of aging.

## Main

**Fig. 1:**
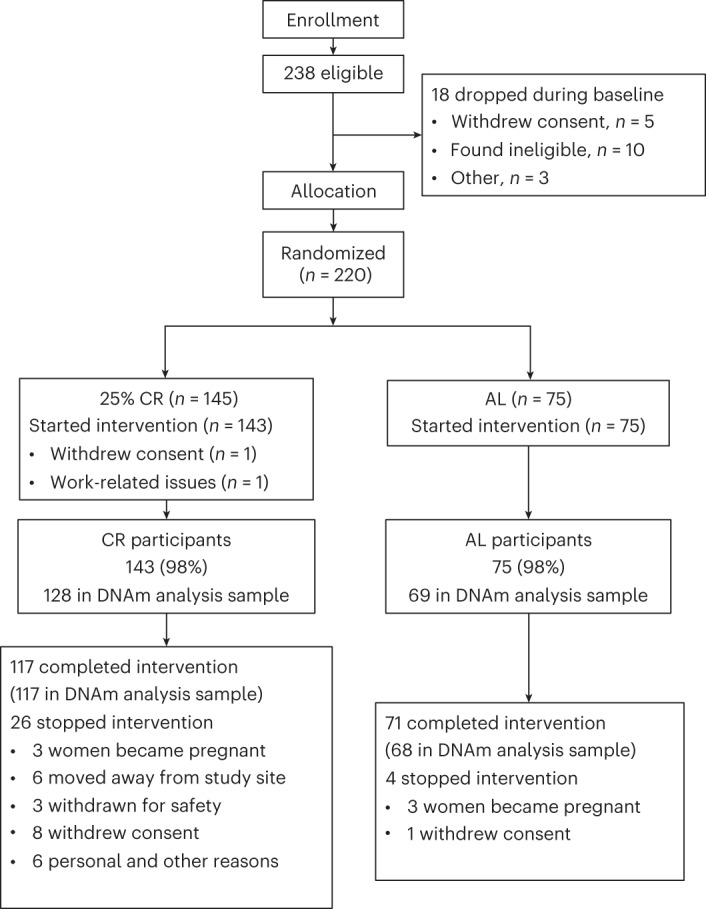
Consort diagram for the CALERIE Trial. DNAm was assayed from blood samples collected at baseline, 12 months and 24 months. Of the *n* = 197 participants for whom DNAm data were available from baseline and at least one follow-up assessment, baseline to 12-month change was measured for *n* = 125 CR and *n* = 66 AL participants and baseline to 24-month change was measured for *n* = 117 CR and *n* = 68 AL participants.

Comprehensive Assessment of Long-term Effects of Reducing Intake of Energy (CALERIE) Phase 2 was a multi-center, randomized controlled trial conducted at three clinical centers in the United States^10^. It aimed to evaluate the time-course effects of 25% CR (that is, intake 25% below the individual’s baseline level) over a 2-yr period in healthy adults (men aged 21–50 yr, premenopausal women aged 21–47 yr) with body mass index (BMI) in the normal weight or slightly overweight range (BMI 22.0–27.9 kg m^−2^). Participants were randomly assigned at a ratio of 2:1 to a CR behavioral intervention or to an ad libitum (AL) control group stratified by site, sex and BMI. Of 238 eligible individuals, CALERIE randomized *N* = 220 participants (145 CR intervention and 75 AL control; Fig. 1). Participants in the CR group were prescribed a 25% restriction in calorie intake based on energy requirements estimated from two 2-week doubly labeled water (DLW) measurement periods at baseline. The precise level of CR achieved was quantified by comparing energy intake (determined periodically throughout the trial by the DLW method^21^) during the CR intervention with baseline energy intake. The CALERIE Trial is described in more detail in Methods.

Blood DNAm data were generated at baseline and at least one follow-up timepoint for *n* = 197 participants (128 CR and 69 AL). Of this analysis sample, *n* = 105 (82%) CR participants and *n* = 59 (86%) AL participants had DNAm data available from all three timepoints (baseline, 12 months and 24 months). DNAm analysis is described in more detail in Methods. Participants had a mean age of 38 yr (s.d. = 7), 70% were women and 77% were white; there were no differences in age, sex or race/ethnicity between AL and CR at baseline (Table 1).

**Table 1:** Characteristics of CALERIE Trial participants at baseline

	AL		CR	
	*N*/mean	(%/s.d.)	*N*/mean	(%/s.d.)
**CALERIE participant characteristics at baseline**				
Age at baseline (yr)	38	(7)	38	(7)
Energy intake (kcal d^−1^)	2,045	(481)	2,124	(558)
Sex				
Women	53	(71%)	100	(69%)
Men	22	(29%)	45	(31%)
Race/ethnic identity				
White	57	(76%)	111	(77%)
Black	11	(15%)	16	(11%)
Other	7	(9%)	18	(12%)
Study site				
A	25	(33%)	47	(32%)
B	27	(36%)	53	(37%)
C	23	(31%)	45	(31%)
BMI stratum				
22.0–24.9 kg m^−2^	37	(54%)	70	(48%)
25.0–27.9 kg m^−2^	38	(55%)	75	(52%)
**Analysis sample characteristics at baseline**				
Age at baseline	38	(7)	38	(7)
Energy intake (kcal d^−1^)	2,023	(469)	2,130	(540)
Sex				
Women	48.00	(70%)	89	(70%)
Men	21.00	(30%)	39	(30%)
Race/ethnic identity				
White	52.00	(75%)	100	(78%)
Black	10	(14%)	13	(10%)
Other	7	(10%)	15	(12%)
Study site				
A	24	(35%)	42	(33%)
B	25	(36%)	45	(35%)
C	20	(29%)	41	(32%)
BMI stratum				
22.0–24.9 kg m^−2^	33	(48%)	60	(47%)
25.0–27.9 kg m^−2^	36	(52%)	68	(53%)

The table shows data for participants randomized to the AL control group and the CR treatment group. CALERIE included a total of *N* = 220 participants (AL *n* = 75, of whom *n* = 71 completed the study; CR *n* = 145, of whom *n* = 118 completed the study). The analysis sample was composed of CALERIE participants for whom DNAm data were available at baseline and at least one follow-up assessment (‘analysis sample’; *N* = 197; AL *n* = 69, CR *n* = 128).

The goal of our analysis was to test the effect of CALERIE intervention on biological aging. We measured biological aging from blood DNAm using published algorithms. These algorithms aim to capture the accumulation of molecular changes that underlie the progressive loss of system integrity that occurs with advancing chronological age. Primary analysis focused on the PhenoAge^22^ and GrimAge^23^ second-generation DNAm clocks and the DunedinPACE^24^ measure of pace of aging, all of which show strong associations with aging-related morbidity and mortality. We analyzed versions of the PhenoAge and GrimAge clocks constructed from DNAm principal components (PCs) (hereafter ‘PC clocks’), which have superior technical reliability as compared with the original versions of these measures^25^; DunedinPACE was originally designed to have high technical reliability. Measures are described in detail in Table 2 and Methods. Associations of DNAm measures of aging with chronological age at preintervention baseline are shown in Supplementary Fig. 2. Mean values of the DNAm measures of aging in the CR and AL groups at baseline and each follow-up are reported in Supplementary Table 1. Intraclass correlation coefficients for tests of technical reliability and within-individual stability are reported in Supplementary Table 2.

**Table 2:** DNAm clock and pace-of-aging measures included in CALERIE analysis

**DNAm clocks**. DNAm clock measures of aging are algorithms that estimate biological age, the state of an organism’s biology represented as the age at which that state would be typical in a reference population. The clocks we analyzed were developed to predict mortality risk. The age values computed by the clock algorithms correspond to the age at which predicted mortality risk would be approximately normal in the reference population used to develop the clock. We computed clock values based on versions of the clock algorithms developed from DNAm PCs (sometimes referred to as ‘PC clocks’)^18,21^.	
PhenoAge clock	Based on analysis of nine blood chemistry markers, age and mortality data from the US National Health and Nutrition Examination Surveys (*n* = 9,926 participants aged 18 yr and older; 23 yr of mortality follow-up); DNAm and blood chemistry data from the InCHIANTI Study (*n* = 912 participants aged 21–100 yr); and the US Health and Retirement Study (*n* = 3,593 participants aged 51–100 yr)^19^.
GrimAge clock	Based on analysis of eight plasma protein markers, smoking pack years, age, sex and mortality data from the Framingham Heart Study Offspring Cohort (*n* = 2,356 participants aged 24–92 yr)^47–49^.
**Pace of aging**. Pace-of-aging measures estimate the rate of biological aging, defined as the rate of decline in overall system integrity. Pace-of-aging values correspond to the years of biological aging experienced during a single calendar year. A value of 1 represents the typical pace of aging in a reference population; values above 1 indicate faster pace of aging; values below 1 indicate slower pace of aging.	
DunedinPACE	Based on analysis of pace of aging in the Dunedin Study (*n* = 817 participants examined at ages 26, 32, 38 and 45 yr)^24^. Pace of aging was measured from within-person change over time in 19 blood chemistry and organ function test metrics of system integrity^24^. DNAm was measured at age 45 yr.

**Fig. 2:**
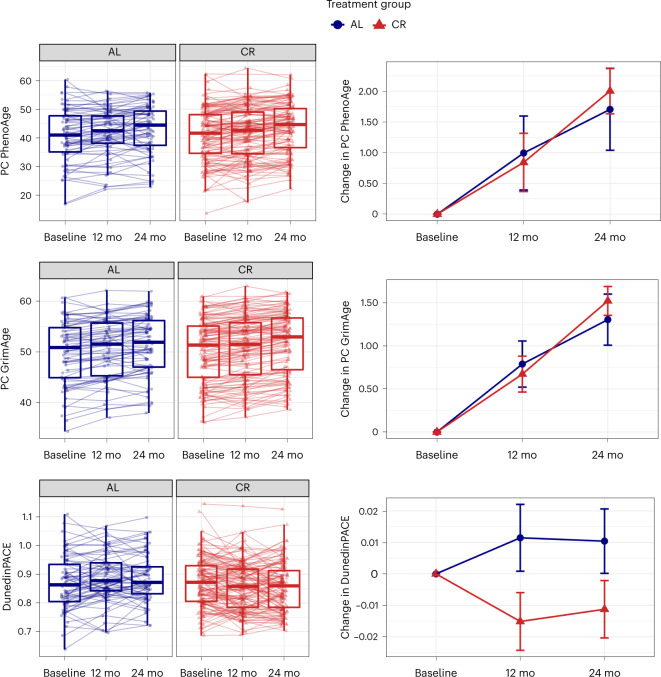
Change from baseline to 12- and 24-month follow-up in DNAm measures of aging in the AL and CR groups in the CALERIE Trial. The figure shows CALERIE Trial treatment effects on three DNAm measures of aging, the PC PhenoAge clock, the PC GrimAge clock and DunedinPACE. Values for the AL control group (*n* = 69 participants) are graphed in blue. Values for the CR treatment group (*n* = 128 participants) are graphed in red. For the PhenoAge and GrimAge DNAm clocks, values are denominated in ‘years’ of DNAm age. For the clocks, expected change under the null hypothesis is 1 yr at 12-month follow-up and 2 yr at 24-month follow-up. For the DunedinPACE measure, values are denominated in pace-of-aging units scaled to be interpretable as percentage difference in the rate of aging relative to the reference norm of 1 yr of biological decline per calendar year. For DunedinPACE, expected change under the null hypothesis is zero. The left column of the figure shows box plots of the observed values of the measures at baseline and 12- and 24-month follow-ups. The boxes show the interquartile range; the whiskers show 1.5× the interquartile range; the center line shows the median; individual participant data are plotted as dots and connected with lines. For the PC PhenoAge and PC GrimAge DNAm clocks, the box plots show similar patterns of increase in both AL and CR groups. For DunedinPACE, the box plot shows stability in the AL group and decrease in the CR group. The right column of the figure shows mean values of change from baseline and 95% CIs estimated from mixed models at the 12- and 24-month follow-ups for the AL and CR groups. There is no confidence interval estimated for baseline because change from baseline is exactly zero at this timepoint. For the PC PhenoAge and PC GrimAge DNAm clocks, mean change is similar in the AL and CR groups. For DunedinPACE, mean change is positive in the AL group (although the confidence interval overlaps zero at 24 months) and negative in the CR group. mo, months. Source data

We computed change scores for the DNAm measures of aging as the differences of 12-month and 24-month follow-up values from baseline values. For analysis, change scores were scaled so that effect sizes can be interpreted as standardized differences between means (Cohen’s *d*). For PhenoAge and GrimAge clocks, change score values were scaled by the standard deviation of the difference between clock age and chronological age at pretreatment baseline. For DunedinPACE, which measures pace of aging (that is, change in biological age per chronological year), change score values were scaled by the standard deviation at pretreatment baseline. Scaled change scores are reported in Supplementary Table 3. Change scores are graphed in Fig. 2 and Supplementary Fig. 3.

To test the hypothesis that CR slowed biological aging, we conducted intent-to-treat (ITT) analysis which compared change scores between participants randomized to CR intervention and AL control group using repeated-measures analysis of covariance (ANCOVA) implemented under mixed models, following the approach used in past CALERIE analysis^26^. Model details are reported in Methods. We use *P* < 0.005 as a conservative threshold for statistical significance following guidance from leaders in the field^27^. As expected, participants’ PhenoAge and GrimAge values tended to increase over time. However, change in PhenoAge and GrimAge values did not differ between CR and AL groups (for PhenoAge, 12-month *d* = −0.03 (95% confidence interval (95% CI) −0.19, 0.12), 24-month *d* = 0.05 (95% CI −0.11, 0.20), *P* > 0.50 for both; for GrimAge, 12-month *d* = −0.04 (95% CI −0.16, 0.07), 24-month *d* = 0.05 (95% CI −0.07, 0.17), *P* > 0.40 for both). CR treatment reduced participants’ DunedinPACE by the 12-month follow-up and this reduction was maintained through follow-up at 24 months (12-month *d* = −0.29 (95% CI −0.45, −0.13), 24-month *d* = −0.25 (95% CI −0.41, −0.09), *P* < 0.003 for both). Standardized treatment effects on DunedinPACE correspond to a reduction in the pace of aging of 2–3%. These average treatment effects summarize diverse responses to intervention; for some treatment group participants, reductions in DunedinPACE were much larger, whereas, for others, DunedinPACE increased from baseline to follow-up. ITT results are reported in Supplementary Table 4.

In the CALERIE Trial, the %CR achieved by participants in the treatment group varied, with most participants achieving doses below the prescribed 25% (mean = 11.9, s.e.m. = 0.7%)^10^. We therefore conducted analyses (1) to test if those who achieved higher CR doses experienced larger treatment effects (dose–response); and (2) to quantify the treatment effect that would be expected among individuals achieving a high dose (we selected a dose of 20%, the 75th percentile of the CR distribution in the treatment group at 12 months, hereafter ‘effect-of-treatment-on-the-treated’ or TOT). To test dose–response, we stratified CR treatment group participants according to whether they achieved at least 10% CR and repeated ITT analysis. For DunedinPACE, the treatment effect in the >10% CR group was *d* = −0.33 at 12 months and *d* = −0.33 at 24 months, as compared with *d* = −0.19 at 12 months and *d* = −0.14 at 24 months in the <10% CR group. There was no evidence of a dose–response effect for PhenoAge or GrimAge. Full results are reported in Supplementary Table 5. To test the TOT, we conducted instrumental variables (IV) analysis. IV analysis assumes that CALERIE intervention affected participants’ biological aging only through its effect on their caloric intake. Our IV analysis estimated the % reduction in caloric intake each participant achieved because of the intervention and applied these estimates to quantify the effect of %CR on biological aging. In IV analysis, the effect of 20% CR on DunedinPACE was *d* = −0.43 (95% CI −0.67, −0.19) at 12 months and *d* = −0.40 (95% CI −0.67, −0.12) at 24 months (*P* < 0.005 for both). IV effect-size estimates for PhenoAge and GrimAge were small (*d* = −0.13–0.01; *P* > 0.15). TOT results are reported in Supplementary Table 4.

We tested sensitivity of results to changes in white blood cell populations in response to CALERIE intervention by including covariates in our models for DNAm estimates of cell counts^28^; these results were similar to unadjusted analyses (Supplementary Table 6).

We tested sex differences in treatment effects. We repeated ITT and TOT analyses with the addition of a product term testing interaction between the treatment variable and participant sex. Sex differences in treatment effects were not statistically different from zero in any of the models. Means of DNAm measures of aging are reported separately for men and women in Supplementary Tables 7 and 8. Sex-stratified treatment effects and tests of sex differences in treatment effects are reported in Supplementary Tables 9 and 10.

Previous studies have considered a broader set of DNAm measures of aging. In the interest of comparability across studies, we report results for so-called ‘first-generation’ clocks developed to predict chronological age and the original versions of the PhenoAge and GrimAge clocks in the Supplementary Information.

CR effects varied across the DNAm measures of aging we analyzed. CALERIE intervention slowed pace of aging as measured by DunedinPACE, whereas the CR intervention did not affect the PhenoAge and GrimAge DNAm clocks. All three measures have evidence for validity as biomarkers of aging, in particular, evidence of association with aging-related morbidity and mortality and with exposures associated with shortened healthy lifespan^24,29,30^. However, these DNAm measures were developed using different methods and reflect different models of aging. The PhenoAge and GrimAge clocks were developed to predict mortality risk at a single timepoint in mixed-age and older adults. This approach quantifies aging as a static construct of risk accumulated across the lifetime. In contrast, DunedinPACE was developed to predict multi-system physiological decline over two decades of follow-up from early adulthood to midlife. This approach quantifies aging as a dynamic construct reflecting change in risk accumulation. DunedinPACE may therefore be more sensitive than PhenoAge and GrimAge to changes induced by 2 yr of CALERIE intervention.

Our previous reports on CALERIE establish that CR intervention improved participants’ cardiometabolic health and slowed aging-related changes in physiological system integrity^26,31,32^. In some cases, these effects are larger than the effects we observed for DunedinPACE (for example, *d* = 0.2–0.3 for DunedinPACE as compared with *d* = 0.2–0.4 for blood chemistry measures of biological age^32^). Changes in DunedinPACE in response to CR intervention mediated only small fractions of CR-induced changes in clinical measures (Supplementary Fig. 4). The purpose of DNAm analysis in CALERIE was to evaluate intervention effects at the molecular level, where aging processes are posited to originate^33^. Studies in subsets of CALERIE participants suggest effects of CR on molecular mechanisms of immune and metabolic regulation^34,35^. DunedinPACE findings broaden evidence of molecular changes in response to CR to a DNAm biomarker of aging established to predict morbidity and mortality.

Follow-up in the CALERIE Trial did not extend beyond the intervention. It is therefore unclear if the changes in DunedinPACE observed during the 2-yr intervention will translate into reduced morbidity and mortality over the long term. In observational studies with long-term follow-up, individuals with slower DunedinPACE are better-off on a range of healthspan metrics, including showing reduced incidence of morbidity and increased survival^24,29^. These previous studies suggest that the CALERIE treatment effect of 2–3% slower pace of aging corresponds to a reduction in mortality risk of as much as 10–15%, similar in magnitude to the effect of smoking cessation intervention^36^. Additional follow-up of trial participants is required to determine whether CR-induced reductions to DunedinPACE in CALERIE will translate into disease prevention and increased healthy lifespan. Moreover, changes in DunedinPACE over follow-up showed substantial overlap between the CR treatment group and the AL control group; effect-size estimates imply close to 90% overlap of DunedinPACE trajectories between the two groups.

We acknowledge limitations. There is no gold standard measure of biological aging^37^. We analyzed several measures which represent the current state-of-the-art in DNAm quantification of biological aging^38^. Nevertheless, these measures are acknowledged to be incomplete summaries of biological changes that occur with aging and to have technical limitations^39,40^. Treatment effects on aspects of biological aging not captured by the DNAm measures are not included in effect estimates; measurement error due to technical limitations of DNAm assays may bias effect estimates towards the null. Treatment effect estimates may therefore represent a lower-bound of the true impact of CALERIE intervention on biological aging. The measures we studied summarize biological aging in general and do not isolate system-specific aging processes^41^. However, CR has diverse effects across multiple biological systems^42,43^. Our general measures of biological aging thus provide a reasonable test of cross-system impacts. On average, trial participants did not achieve the prescribed dose of 25% CR and some control group participants reduced their caloric intake. Despite this imperfect adherence, treatment group participants experienced substantial and sustained weight loss and related changes in body and tissue composition, broad improvement in cardiometabolic health and a slowing of aging-related physiological changes^26,31,44,45^. Our dose–response and TOT analyses indicated that participants who achieved higher doses of CR experienced more pronounced reductions in DunedinPACE. The CALERIE Trial sample does not represent the general population and treatment effects may not generalize beyond the population of healthy volunteers recruited to participate. CALERIE follow-up is, so far, limited to the end of the intervention period. Whether treatment and any slowing in biological aging that resulted from it translated to long-term clinical benefit is currently unknown.

Within the context of these limitations, our findings have implications for future geroscience research. Aging biology research has identified multiple therapies with potential to improve healthy lifespan in humans. A barrier to advancing translation of these therapies through human trials is that intervention studies run for months or years, but human aging takes decades to cause disease^46–48^. New measurements that summarize biological changes occurring with aging have potential to overcome this challenge; measurements to quantify biological aging that both predict future disease, disability and mortality and can detect changes in aging processes over short timescales have potential to function as surrogate endpoints for intervention effects on healthy lifespan^38,49^. The methods proposed to quantify biological aging analyzed in this study are predictive of aging-related health decline and mortality. However, until this study, none had been tested in a randomized controlled trial of a geroscience-based intervention^49^. Our findings highlight DunedinPACE as a measure with potential utility in future trials. DunedinPACE has high test–retest reliability and shows strong associations with healthspan endpoints in validation analyses^24,29^. Ultimately, establishing DunedinPACE and other DNAm measures of aging as surrogate endpoints for geroscience will require evidence that changes in DNAm measures account for intervention effects on primary healthy-aging endpoints, including incidence of chronic disease and mortality^18–20^. The evidence reported from CALERIE suggests that DunedinPACE may be helpful in identifying short-term interventions worthy of long-term follow-up to generate such evidence.

CALERIE was a 24-month, intensive behavioral intervention to deliver a therapy proven to slow aging in animal models. Although treatment effect sizes were small, even modest slowing of the pace of aging can have profound effects on population health^11–13^. Future trials, especially those considering less-intensive or shorter-term interventions, such as intermittent fasting^50^, should plan for larger samples to ensure adequate statistical power. Further, efforts to forecast potential benefits from interventions designed to delay aging may best serve policy makers and planners if they work from assumptions of modest intervention effects.

## Methods

We conducted new DNAm assays of stored blood biospecimens collected from the CALERIE Phase 2 randomized controlled trial and merged these data with existing secondary data from the trial. The assays of the biospecimens were conducted blind to the conditions of the trial. Details of trial design and the collection of other trial data were reported previously^10,26^.

### Study design and participants

CALERIE Phase 2 was a multi-center, randomized controlled trial conducted at three clinical centers in the United States^10^ (ClinicalTrials.gov Identifier: NCT00427193). It aimed to evaluate the time-course effects of 25% CR (that is, intake 25% below the individual’s baseline level) over a 2-yr period in healthy adults (men aged 21–50 yr, premenopausal women aged 21–47 yr) with BMI in the normal weight or slightly overweight range (BMI 22.0–27.9 kg m^−2^). The study protocol was approved by Institutional Review Boards at three clinical centers (Washington University School of Medicine, St Louis, MO, USA; Pennington Biomedical Research Center, Baton Rouge, LA, USA; Tufts University, Boston, MA, USA) and the coordinating center at Duke University (Durham, NC, USA). All study participants provided written, informed consent. Nongenomic data were obtained from the CALERIE Biorepository (https://calerie.duke.edu/apply-samples-and-data-analysis).

### Randomization and masking

After baseline testing, participants were randomly assigned at a ratio of 2:1 to a CR behavioral intervention or to an AL control group. Randomization was stratified by site, sex and BMI. A permuted block randomization technique was used.

### Procedures

Study procedures were published previously^10,21,26^ and are described here in brief. Participants in the CR group were prescribed a 25% restriction in calorie intake based on energy requirements estimated from two DLW measurement periods at baseline. Participants were provided three meals per day for 27 d to familiarize themselves with portion sizes for a 25% reduced calorie intake; meals included eating plans modified to suit various cultural preferences. Participants also received instruction on the essentials of CR. Finally, participants were provided with intensive group and individual behavioral counseling sessions once a week, with 24 group and individual counseling sessions over the first 24 weeks of the intervention. Adherence to the CR intervention was estimated in real time by the degree to which individual weight change followed a predicted weight loss trajectory (15.5% weight loss at 1 yr followed by weight loss maintenance). The precise level of CR achieved was quantified retrospectively by calculating energy intake during the CR intervention and comparing it with baseline energy intake. Energy intake during the 2-yr trial was quantified from total daily energy expenditure (assessed during 2-week DLW periods every 6 months) and changes in body composition (that is, fat mass and fat-free mass). Participants assigned to the AL group continued on their regular diets; they received no specific dietary intervention or counseling. They had quarterly contact with study investigators to complete the assessments.

### Quantification of %CR

Mean %CR was calculated at each of the follow-up timepoints as percentage decrease in energy intake relative to baseline using the equation %CR_mean_ = (1 − EI_mean_/EI_BL_) × 100 (ref. ^21^). EI_BL_ was defined as total energy expenditure (TEE) at preintervention baseline and EI_mean_ was defined as the average of TEE across all follow-up visits through the visit at which %CR was calculated. TEE was measured by the DLW method during two consecutive 2-week periods at baseline and during 2-week periods at months 6, 12, 18 and 24 in the CR group^10,44^.

### DNAm data

DNA extracted from blood samples was obtained from the CALERIE Biorepository at the University of Vermont. DNAm data were generated by the Kobor Lab at the University of British Columbia and processed by the Genomic Analysis and Bioinformatics Shared Resource at Duke University. Illumina Infinium Methylation EPIC BeadChip arrays were used to assay genome-wide DNAm data from banked DNA samples extracted from blood collected at the baseline, 12-month and 24-month follow-ups. The EPIC array quantifies DNAm levels at >850,000 CpG sites across all known genes, regions and key regulatory regions. Briefly, 750-ng extracted DNA samples were bisulfite converted using the EZ DNA Methylation kit (Zymo Research), and 160 ng of the converted DNA was used as input for the EPIC arrays (Illumina). EPIC arrays were processed according to the manufacturer’s instructions and scanned using the Illumina iScan platform. To the extent possible, baseline, 12-month and 24-month samples from the same individual were processed in the same array batch and on the same BeadChip to minimize batch effects; CR treatment and AL control participants were included on all chips. Quality control and normalization analyses were performed using the methylumi (v.2.32.0)^51^ Bioconductor (v.2.46.0)^52^ package for the R statistical programming environment (v.3.6.3). Probes were considered missing in a sample if they had detection *P* values >0.05 and were excluded from the analysis if they were missing in >5% of sample. Normalization to eliminate systematic dye bias in 2-channel probes was carried out using the methylumi default method. Following quality control and normalization, DNAm data for 828,613 CpGs were available for *n* = 595 samples (baseline *n* = 214; 12 months *n* = 193; 24 months *n* = 188). Additional batch correction was performed by residualizing DNAm measurements for PCs estimated from array control-probe beta values^53^. Cell count estimation was performed using the Houseman equation via the *minfi* and *FlowSorted.Blood.EPIC* R packages^28,54^.

### DNAm clocks and pace-of-aging measures

DNAm clocks are algorithms that combine information from DNAm measurements across the genome to quantify variation in biological age^55^.

The first-generation DNAm clocks were developed from machine-learning analyses comparing samples from individuals of different chronological age. These clocks were highly accurate in predicting the chronological age of new samples and also showed some capacity for predicting differences in mortality risk, although effect sizes tend to be small and inconsistent across studies^56–58^. We analyzed the first-generation clocks proposed by Horvath (Horvath clock) and Hannum et al. (Hannum clock)^56,57^.

The second-generation DNAm clocks were developed with the goal of improving quantification of biological aging by focusing on differences in mortality risk instead of on differences in chronological age^22,23^. These clocks also include an intermediate step in which DNAm data are fitted to physiological parameters. The second-generation clocks are more predictive of morbidity and mortality as compared with the first-generation clocks^59^ and are proposed to have improved potential for testing impacts of interventions to slow aging^14^. We analyzed the second-generation clocks proposed by Levine et al. (PhenoAge clock) and Lu et al. (GrimAge clock)^22,23^.

A limitation of several DNAm clocks is that when residualized for chronological age, values show only moderate test–retest reliability across technical replicates. Test–retest reliability is a critical feature of measurements used to evaluate the impact of intervention because change from preintervention to postintervention cannot be distinguished from technical noise unless reliability is high. To improve technical reliability, Higgins-Chen and colleagues developed a new computational method that retrained DNAm clocks using DNAm PCs^25^. The resulting ‘PC clocks’ demonstrate exceptional test–retest reliability across technical replicates.

A third generation of DNAm measures of aging are referred to as pace-of-aging measures. In contrast to first- and second-generation DNAm clocks, which aim to quantify how much aging has occurred up to the time of measurement, pace-of-aging measures aim to quantity how fast the process of aging-related deterioration of system integrity is proceeding. We analyzed the newest pace-of-aging measure, DunedinPACE, which is shorthand for ‘Pace of Aging Computed from the Epigenome’^24^. DunedinPACE was developed by modeling within-individual multi-system physiological change across four timepoints in same-age individuals in the Dunedin Study 1972–1973 birth cohort^60,61^, when participants were aged 26, 32, 38 and 45 yr. DunedinPACE was developed from analysis of a pace-of-aging composite of slopes of aging-related change in the following physiological measures: ApoB100/ApoA1 ratio, BMI, blood urea nitrogen, high-sensitivity C-reactive protein, cardiorespiratory fitness, dental caries experience, total cholesterol, forced expiratory volume in 1 second, forced expiratory volume in 1 second/fixed vital capacity ratio, estimated glomerular filtration rate, hemoglobin A1C, high-density lipoprotein cholesterol, leptin, lipoprotein(a), mean arterial pressure, mean periodontal attachment loss, triglycerides, waist-to-hip ratio and white blood cell count. Slopes of change were estimated from four repeated measurements collected over a period of two decades. This physiological pace-of-aging composite is described in detail in ref. ^61^. The DunedinPACE DNAm algorithm was derived from elastic net regression of the physiological pace-of-aging composite on Illumina EPIC array DNAm data derived from blood samples collected at the age 45 follow-up assessment. The set of CpG sites included in the DNAm dataset used to develop the DunedinPACE algorithm was restricted to those showing acceptable test–retest reliability as determined in the analysis in ref. ^62^. The DunedinPACE DNAm algorithm is described in detail in ref. ^24^.

Our primary analysis focused on the PC versions of the PhenoAge and GrimAge second-generation clocks and DunedinPACE, all of which show exceptional test–retest reliability in technical replicates. We report results for both original and PC versions of DNAm clocks in the Supplementary Information.

### Analysis

Analysis included all participants with available DNAm data at trial baseline and at least one follow-up timepoint.

We computed change scores for all aging measures by comparing values at the 12-month and 24-month follow-up assessments with baseline values (that is, 12-month change = 12-month value − baseline; 24-month change = 24-month value − baseline). We conducted analyses of these change scores to test the hypothesis that CR slows biological aging using two complementary approaches: (1) we conducted ITT analysis which compared change scores between participants randomized to CR intervention and the AL control group; (2) we conducted TOT analysis using IV methods to estimate the effect of CR on change scores.

In ITT analysis, we tested the effect of randomization to CR versus AL on aging measure change scores using repeated-measures ANCOVA implemented under mixed models, following the approach used in past CALERIE analysis^26^. The model included terms for treatment condition (CR or AL), follow-up time, an interaction term modeling heterogeneity in the treatment effect between the 12- and 24-month follow-ups, the baseline level of the aging measure and the following pretreatment covariates: chronological age, sex, race/ethnicity (Black, White, Other), BMI stratum at randomization (normal weight (22.0–24.9 kg m^−2^) and overweight (25.0–27.9 kg m^−2^)) and study site. Models were fitted using the Stata software’s ‘mixed’ command. Details of estimation and calculation of confidence intervals are reported in Stata’s documentation of the command^63^.

In TOT analysis, we tested the effect of the CR intervention on aging measure change scores using IV regression implemented using a two-stage least squares approach^64^. The first-stage regression modeled CR treatment dose as a function of randomization condition (CR versus AL) and pretreatment characteristics (chronological age, sex, race/ethnicity, BMI, study site and baseline value of the biological aging measure). The model instruments were randomization condition and interactions of randomization condition with sex and pretreatment values of BMI and the biological aging measure. The second-stage regression modeled aging measure change scores as a function of the CR treatment dose estimated from the first-stage regression and pretreatment covariates. Separate models were fitted for the 12- and 24-month follow-ups. IV regression models were fitted using the Stata 16.0 software’s ‘ivregress’ command. Details of estimation and calculation of confidence intervals are reported in Stata’s documentation of the command^65^. TOT models are described in detail below.

In ITT and TOT analyses, effect sizes were scaled in standardized units according to the distribution of the aging measures at pretreatment baseline. For the DNAm clocks, clock ages were differenced from chronological ages and standard deviations for these age-difference values were used for scaling. For DunedinPACE, the standard deviations of the original values were used for scaling. Treatment effects denominated in these standardized units are interpreted as Cohen’s *d*.

### Specification of TOT regression models

We tested TOT effects using two-stage least squares IV regression. IV regression is a method commonly used to reduce the impact of confounding in association analysis. It can also be applied to account for contamination/nonadherence in randomized trials^64^. Under conditions of nonadherence, traditional ITT analysis can result in a biased estimate of the treatment effect and an IV estimator can provide a complement^66^. In CALERIE, adherence was imperfect; the average CR achieved in the treatment group was roughly half the prescribed dose of 25% (ref. ^10^). The ITT estimate may therefore underestimate the effect of CR on biological aging.

In our analysis, we used IV regression to estimate the effect of 20% CR on change in measures of biological aging. We focused on a CR dose of 20% instead of the 25% dose prescribed in the trial because few individuals achieved 25% CR, especially through the 24-month follow-up. The 20% CR level represented the 75th percentile of the treatment group CR distribution at 12-month follow-up and the 87th percentile of the treatment group CR distribution at 24-month follow-up.

The IV approach we used involved two related regressions. The first regression modeled observed treatment dose (%CR relative to baseline) on pretreatment characteristics and the instrument of randomization condition. The second regression modeled the outcomes (changes in measures of biological aging) as functions of the predicted treatment dose estimated by the first regression and pretreatment covariates.

We developed our IV regression model by first modeling intervention group participants’ achieved CR treatment dose as a function of pretreatment covariates: chronological age, sex, BMI, study site. We fitted a saturated regression model including interactions among all pretreatment characteristics and additional covariate adjustment for race/ethnicity, which was included only as a main effect. (Race/ethnicity was omitted from the interaction terms because there was insufficient site- and sex-specific variation in race/ethnicity to fit models.) This analysis identified sex, baseline BMI and their interaction as statistically significant predictors of CR dose at the alpha = 0.05 level.

Next, we parameterized our IV regression specifying the first stage to include the ‘instruments’ of intervention group and interactions of intervention group with sex, pretreatment BMI and a three-way interaction between intervention condition, sex and pretreatment BMI. The base first-stage regression took the form

1 $$\begin{array}{l}\% {{{\mathrm{CR}}}}_{{{t}}} = {{{a}}} + {{{\mathrm{CR}}}} + {{{\mathrm{CR}}}} \times {{{\mathrm{sex}}}} + {{{\mathrm{CR}}}} \times {{{\mathrm{BMI}}}}_{{{{\mathrm{baseline}}}}} \\+ {{{\mathrm{CR}}}} \times {{{\mathrm{sex}}}} \times {{{\mathrm{BMI}}}}_{{{{\mathrm{baseline}}}}} + {{{X}}} + e\end{array}$$

in which %CR_*t*_ is the %CR relative to baseline achieved at time *t* (either 12- or 24-month follow-up), BMI_baseline_ is pretreatment BMI, *X* is a matrix of all pretreatment covariates, *a* is a model intercept and *e* is the error term. Results from this first-stage regression were then included in the second-stage model:

2 $${{{\mathrm{Delta}}}}\,{{{\mathrm{BA}}}}_{{{t}}} = {{{a}}} + \% {{{\mathrm{CR}}}}_{{{t}}} + {{{\mathrm{X}}}} + {{{e}}}$$

in which %CR_*t*_ is %CR predicted from equation (1). For final TOT analysis, we included a further instrument in the first-stage regression consisting of the interaction between the baseline level of the aging measure and the CR treatment group. Sensitivity analysis involving re-estimating the IV regression models omitting this final instrument did not change results.

Supplementary Fig. 1 plots predicted values of %CR based on our base first-stage model (that is, the model in equation (1)).

### Statistics and reproducibility

We conducted new DNAm assays of stored blood biospecimens collected from the CALERIE Phase 2 randomized controlled trial and merged these data with existing secondary data from the trial. The assays of the biospecimens were conducted blind to the conditions of the trial. After baseline testing, *n* = 220 participants were randomly assigned at a ratio of 2:1 to a CR behavioral intervention or to an AL control group. Randomization was stratified by site, sex and BMI. A permuted block randomization technique was used. No statistical methods were used to predetermine sample sizes; we analyzed data from all participants for whom blood DNAm data were available at baseline and at least one follow-up timepoint (*N* = 197; CR *n* = 128, AL *n* = 69). Participants had mean age of 38 yr (s.d. = 7), 70% were women and 77% were white; there were no differences in age, sex or race/ethnicity between AL and CR at baseline (Table 1). Data met model assumptions. Normality of outcome variables was evaluated by visual inspection of distributions and the Shapiro–Wilk test^67^. Equality of variances was evaluated according to the tests proposed by Brown and Forsythe^68^ and Markowski and Markowski^69^. Models used to test ITT and TOT effects were fitted with heteroskedasticity-robust standard errors. Normality of distribution of error terms was evaluated by visual inspection of histograms of residuals and the Shapiro–Wilk test.

### DNAm clocks

DNAm clock measures of aging are algorithms that estimate biological age, the state of an organism’s biology represented as the age at which that state would be typical in a reference population. The clocks we analyzed were developed to predict mortality risk. The age values computed by the clock algorithms correspond to the age at which predicted mortality risk would be approximately normal in the reference population used to develop the clock. We computed clock values based on versions of the clock algorithms developed from DNAm PCs (sometimes referred to as ‘PC clocks’)^18,21^.

#### PhenoAge clock

The PhenoAge clock was based on analysis of nine blood chemistry markers, age and mortality data from the US National Health and Nutrition Examination Surveys (*n* = 9,926 participants aged 18 yr and older; 23 yr of mortality follow-up); DNAm and blood chemistry data from the Invecchiare in Chianti (InCHIANTI) Study (*n* = 912 participants aged 21–100 yr); and the US Health and Retirement Study (*n* = 3,593 participants aged 51–100 yr)^19^.

#### GrimAge clock

The GrimAge clock was based on analysis of eight plasma protein markers, smoking pack years, age, sex and mortality data from the Framingham Heart Study Offspring and Gen3 Cohorts (*n* = 2,751 participants aged 24–92 yr)^47–49^.

### Pace of aging

Pace-of-aging measures estimate the rate of biological aging, defined as the rate of decline in overall system integrity. Pace-of-aging values correspond to the years of biological aging experienced during a single calendar year. A value of 1 represents the typical pace of aging in a reference population; values above 1 indicate faster pace of aging; values below 1 indicate slower pace of aging.

#### DunedinPACE

Based on analysis of pace of aging in the Dunedin Study (*n* = 817 participants examined at ages 26, 32, 38 and 45 yr)^24^, pace of aging was measured from within-person change over time in 19 blood chemistry and organ function test metrics of system integrity^24^. DNAm was measured at age 45 yr.

### Reporting summary

Further information on research design is available in the Nature Portfolio Reporting Summary linked to this article.

## Supplementary information

Supplementary Information Supplementary Tables 1–10, Figs. 1–4 and references.

Reporting Summary

Supplementary Data 1 Clock values, CR treatment group and follow-up data used for Supplementary Fig. 3a.

Supplementary Data 2 Effects estimates of CR treatment from mixed models of change in epigenetic age used in Supplementary Fig. 3b.

## Data Availability

All data, including DNA methylation data, are available for academic research purposes from the CALERIE Biorepository: https://calerie.duke.edu. Instructions for applying for data access are detailed at https://calerie.duke.edu/samples-data-access-and-analysis. Applications for some types of data may require IRB oversight. Guidelines are available at https://calerie.duke.edu/sites/default/files/2022-08/calerie_ancillary_study_guidelines_revised_042921.pdf. Registration to obtain an application form can be completed at https://calerie.duke.edu/database-submission-form. Phenotypic data used in the primary analysis were obtained from the analysis datasets ‘subject1’, ‘visits’, ‘ivrsrand’, ‘clwtvis’ and ‘pctcr’. Additional data were obtained from ‘teerq’, ‘rmrresid’, ‘vitalsa’, ‘oclabflt’. Source data for Fig. 2 and Supplementary Fig. 3 are provided in Supplementary Information.

## Source data

Source Data Fig. 2 Clock values, CR treatment group and follow-up data used for Fig. 2, left column.
Source Data Fig. 2 Effects estimates of CR treatment from mixed models of change in epigenetic age used in Fig. 2, right column.

## Code availability

Code is available from GitHub: https://github.com/danbelsky/CALERIE_ClocksAnalysis.
