# Author Correction: Effect of long-term caloric restriction on DNA methylation measures of biological aging in healthy adults from the CALERIE trial

**DOI:** 10.1038/s43587-023-00432-y

**Published:** 2023-05-09

**Authors:** R. Waziry, C. P. Ryan, D. L. Corcoran, K. M. Huffman, M. S. Kobor, M. Kothari, G. H. Graf, V. B. Kraus, W. E. Kraus, D. T. S. Lin, C. F. Pieper, M. E. Ramaker, M. Bhapkar, S. K. Das, L. Ferrucci, W. J. Hastings, M. Kebbe, D. C. Parker, S. B. Racette, I. Shalev, B. Schilling, D. W. Belsky

**Affiliations:** 1 Butler Columbia Aging Center, Columbia University Mailman School of Public Health, New York, NY USA; 2 Department of Genetics, University of North Carolina at Chapel Hill School of Medicine, Chapel Hill, NC USA; 3 Duke Molecular Physiology Institute and Department of Medicine, Duke University School of Medicine, Durham, NC USA; 4 Department of Medical Genetics, Edwin S.H. Leong Healthy Aging Program, Centre for Molecular Medicine and Therapeutics, University of British Columbia, Vancouver, British Columbia Canada; 5 Department of Epidemiology, Columbia University Mailman School of Public Health, New York, NY USA; 6 Center on Aging and Development, Biostatistics and Bioinformatics, Duke University, Durham, NC USA; 7 Duke Clinical Research Institute, Duke University School of Medicine, Durham, NC USA; 8 Jean Mayer USDA Human Nutrition Research Center on Aging at Tufts University, Boston, MA USA; 9 Translational Gerontology Branch, National Institute on Aging, National Institutes of Health, Baltimore, MD USA; 10 Department of Biobehavioral Health, Pennsylvania State University, State College, PA USA; 11 Pennington Biomedical Research Center, Baton Rouge, LA USA; 12 Department of Medicine, Duke University School of Medicine, Durham, NC USA; 13 Program in Physical Therapy and Department of Medicine, Washington University School of Medicine, St. Louis, MO USA; 14 College of Health Solutions, Arizona State University, Phoenix, AZ USA; 15 Buck Institute for Research on Aging, Novato, CA USA

**Keywords:** Predictive markers, Predictive markers

Correction to: *Nature Aging* 10.1038/s43587-022-00357-y. Published online 9 February 2023.

In the version of this article initially published, the first affiliation listed for D. W. Belsky was incorrect; it should have been shown as Butler Columbia Aging Center, Columbia University Mailman School of Public Health, New York, NY, USA. The error has been corrected in the HTML and PDF versions of the article.

