# Supplementary material for: Effect of long-term caloric restriction on DNA methylation measures of biological aging in healthy adults from the CALERIE trial
Source: Nat Aging. 2023 Feb 9;3(3):248–57. doi: 10.1038/s43587-022-00357-y (PMC10148951; doi:10.1038/s43587-022-00357-y)
Supplement: Supplementary file 1 — Supplementary Tables 1–10, Figs. 1–4 and references. [file 43587_2022_357_MOESM1_ESM.pdf]

nature aging

Letter

https://doi.org/10.1038/s43587-022-00357-y

Effect of long-term caloric restriction on DNA methylation measures of biological aging in healthy adults from the CALERIE trial

In the format provided by the authors and unedited

SUPPLEMENTARY TABLES

Table S1. DNA-methylation clock and Pace of Aging values for CALERIE™ participants in the Ad Libitum (AL) and Caloric Restriction (CR) groups at baseline, 12-month, and 24-month assessments. Panel A shows values for original and PC-based versions[18] of first-generation clocks. Panel B shows values for original and PC-based versions of second-generation clocks. Panel C shows values for DunedinPACE. Values for DNA methylation clocks are age-difference scores computed by subtracting participants' chronological ages from their clock ages. DunedinPACE values are not adjusted for chronological age. 95% Confidence intervals are reported in brackets. Follow-up DNA methylation data for the n=69 AL and n=128 CR participants in the analysis sample were available for n=65 AL and n=125 CR participants at 12-months and n=68 AL and 117 CR participants at 24-months. The positive offset observed for the DNAm clocks is a common feature of observations made using EPIC arrays[27].

Panel A. First-Generation Clocks

		Ad Libitum (n=69)		Calorie Restriction (n=128)	
		**Mean**	**95% CI**	**Mean**	**95% CI**
Horvath Clock	Baseline	-2.08	[-2.94 , -1.22]	-2.06	[-2.75 , -1.38]
	12mo	-2.44	[-3.37 , -1.51]	-2.09	[-2.79 , -1.39]
	24mo	-2.38	[-3.28 , -1.48]	-2.16	[-2.89 , -1.43]
PC Horvath Clock	Baseline	9.06	[8.07 , 10.04]	9.20	[8.50 , 9.90]
	12mo	8.71	[7.66 , 9.77]	9.02	[8.34 , 9.71]
	24mo	8.58	[7.55 , 9.61]	8.74	[8.00 , 9.49]
Hannum Clock	Baseline	3.87	[3.22 , 4.52]	4.17	[3.67 , 4.67]
	12mo	3.59	[2.81 , 4.36]	3.98	[3.45 , 4.50]
	24mo	3.75	[3.05 , 4.46]	3.98	[3.43 , 4.52]
PC Hannum Clock	Baseline	13.44	[12.58 , 14.29]	13.64	[13.04 , 14.24]
	12mo	13.24	[12.28 , 14.19]	13.46	[12.86 , 14.06]
	24mo	13.11	[12.22 , 14.00]	13.29	[12.64 , 13.94]
Skin & Blood Clock	Baseline	1.39	[0.67 , 2.12]	1.60	[1.13 , 2.06]
	12mo	1.73	[1.03 , 2.44]	1.69	[1.24 , 2.15]
	24mo	1.92	[1.16 , 2.69]	1.68	[1.17 , 2.19]
PC Skin & Blood Clock	Baseline	6.44	[5.34 , 7.53]	6.92	[6.16 , 7.69]
	12mo	6.00	[4.80 , 7.19]	6.69	[5.95 , 7.44]
	24mo	5.71	[4.57 , 6.85]	6.31	[5.50 , 7.12]

Table S1 Continued

Panel B. Second-Generation Clocks

		Ad Libitum (n=69)		Calorie Restriction (n=128)	
		**Mean**	**95% CI**	**Mean**	**95% CI**
PhenoAge Clock	Baseline	-10.39	[-11.53 , -9.24]	-10.14	[-11.03 , -9.24]
	12mo	-10.59	[-11.82 , -9.35]	-10.10	[-11.04 , -9.16]
	24mo	-10.99	[-12.12 , -9.86]	-10.12	[-11.00 , -9.24]
PC PhenoAge Clock	Baseline	2.48	[1.36 , 3.61]	2.66	[1.81 , 3.51]
	12mo	2.40	[1.24 , 3.55]	2.36	[1.53 , 3.19]
	24mo	2.23	[1.12 , 3.35]	2.51	[1.67 , 3.36]
GrimAge Clock	Baseline	4.85	[4.14 , 5.56]	4.78	[4.32 , 5.24]
	12mo	4.63	[3.93 , 5.33]	4.33	[3.88 , 4.78]
	24mo	4.25	[3.61 , 4.90]	4.06	[3.59 , 4.54]
PC GrimAge Clock	Baseline	11.51	[10.88 , 12.14]	11.85	[11.34 , 12.35]
	12mo	11.23	[10.53 , 11.93]	11.42	[10.92 , 11.92]
	24mo	10.79	[10.16 , 11.43]	11.08	[10.56 , 11.60]

Panel C. Pace of Aging

Dunedin PACE	Baseline	0.94	[0.92 , 0.96]	0.95	[0.93 , 0.96]
	12mo	0.95	[0.93 , 0.97]	0.93	[0.92 , 0.94]
	24mo	0.95	[0.93 , 0.97]	0.93	[0.92 , 0.95]

Table entries for measurements included in primary analysis (PC PhenoAge, PC GrimAge, DunedinPACE) are highlighted in black text.

Table S2. Technical reliability and within-individual stability of DNAm clocks and DunedinPACE in GSE55763 and the CALERIE™ trial. Panel A shows values for original and PC-based versions[18] of first-generation clocks. Panel B shows values for original and PC-based versions of second-generation clocks. Panel C shows values for DunedinPACE. The left side of the table reports technical (test-retest) reliability across technical replicates from n=36 samples measured in duplicate in GSE55763. The right side of the table reports within-individual stability across repeated measurements at baseline, 12months, and 24 months in the CALERIE™ trial. Reliabilities are calculated as intra-class correlation coefficients (ICCs). Pearson r correlation are also reported.

	Technical Reliability (GSE55763)			Within-Individual Stability (CALERIE™)					
				AL			CR		
	ICC	95% CI		**r**	ICC	95% CI	**r**	ICC	95% CI
**Panel A. First-Generation Clocks**									
Horvath Clock	0.824	[0.68 , 0.97]	Bl-12mo	0.79	0.78	[-0.62 , 2.29]	0.78	0.79	[0.71 , 0.85]
			Bl-24mo	0.74	0.71	[0.57 , 0.81]	0.82	0.83	[0.77 , 0.88]
PC Horvath Clock	0.969	[0.95 , 0.99]	Bl-12mo	0.97	0.97	[0.95 , 0.98]	0.96	0.96	[0.94 , 0.97]
			Bl-24mo	0.97	0.96	[0.93 , 0.97]	0.97	0.96	[0.94 , 0.97]
Hannum Clock	0.853	[0.74 , 0.96]	Bl-12mo	0.84	0.83	[0.74 , 0.89]	0.79	0.78	[0.71 , 0.84]
			Bl-24mo	0.82	0.82	[0.72 , 0.88]	0.80	0.79	[0.72 , 0.85]
PC Hannum Clock	0.986	[0.98 , 0.99]	Bl-12mo	0.95	0.95	[0.91 , 0.97]	0.91	0.91	[0.88 , 0.94]
			Bl-24mo	0.92	0.92	[0.87 , 0.95]	0.93	0.93	[0.90 , 0.95]
Skin & Blood Clock	0.882	[0.75 , 1.01]	Bl-12mo	0.83	0.83	[0.73 , 0.89]	0.76	0.76	[0.68 , 0.83]
			Bl-24mo	0.82	0.81	[0.71 , 0.88]	0.85	0.85	[0.79 , 0.89]
PC Skin & Blood Clock	0.978	[0.96 , 0.99]	Bl-12mo	0.96	0.96	[0.93 , 0.97]	0.95	0.95	[0.93 , 0.96]
			Bl-24mo	0.95	0.94	[0.90 , 0.96]	0.96	0.95	[0.93 , 0.97]
**Panel B. Second-Generation Clocks**									
PhenoAge Clock	0.727	[0.55 , 0.91]	Bl-12mo	0.74	0.75	[0.62 , 0.84]	0.74	0.74	[0.65 , 0.81]
			Bl-24mo	0.62	0.62	[0.45 , 0.75]	0.81	0.81	[0.74 , 0.87]
PC PhenoAge Clock	0.987	[0.98 , 1.00]	Bl-12mo	0.85	0.85	[0.77 , 0.91]	0.82	0.82	[0.76 , 0.87]
			Bl-24mo	0.79	0.80	[0.69 , 0.87]	0.89	0.89	[0.84 , 0.92]
GrimAge Clock	0.96	[0.93 , 0.99]	Bl-12mo	0.91	0.92	[0.87 , 0.95]	0.83	0.80	[0.72 , 0.85]
			Bl-24mo	0.87	0.85	[0.77 , 0.91]	0.86	0.80	[0.72 , 0.86]
PC GrimAge Clock	0.988	[0.97 , 1.00]	Bl-12mo	0.93	0.91	[0.86 , 0.95]	0.91	0.86	[0.81 , 0.90]
			Bl-24mo	0.87	0.83	[0.74 , 0.89]	0.93	0.88	[0.83 , 0.92]
**Panel C. Pace of Aging**									
DunedinPACE	0.964	[0.93 , 1.00]	Bl-12mo	0.85	0.84	[0.75 , 0.90]	0.77	0.75	[0.67 , 0.82]
			Bl-24mo	0.84	0.83	[0.74 , 0.89]	0.80	0.79	[0.71 , 0.85]

Table entries for measurements included in primary analysis (PC PhenoAge, PC GrimAge, DunedinPACE) are highlighted in black text.

Table S3. Change scores for DNA-methylation measures of aging at 12-and 24-month follow-ups in CALERIE™-Trial Ad Libitum and Caloric Restriction group participants. Panel A shows change scores for original and PC-based versions[18] of first-generation clocks. Panel B shows change scores for original and PC-based versions of second-generation clocks. Panel C shows change scores for DunedinPACE. The table shows change-scores computed relative to baseline for the aging measures at 12- and 24-month follow-ups in the AL and CR groups. Change scores are scaled in units of the aging measures' standard deviations at pre-treatment baseline. Change scores can be back-converted into the original units of the measures by multiplying by the standard deviation. The standard deviations are as follows: Horvath Clock = 3.84; PC Horvath Clock = 4.07; Hannum Clock = 2.85; PC Hannum Clock = 3.50; Skin & Blood Clock = 2.81; PC Skin & Blood Clock = 4.46; PhenoAge Clock = 5.04; PC PhenoAge Clock = 4.84; GrimAge Clock = 2.76; PC GrimAge Clock = 2.82; DunedinPACE = 0.09. For the clocks, change scores are computed from age-difference values (i.e. clock age – chronological age) at baseline and the 12- and 24-month follow-ups.

		Ad Libitum (n=69)		Calorie Restriction (n=128)	
		**Mean**	**95% CI**	**Mean**	**95% CI**
**Panel A. First-Generation Clocks**					
Horvath Clock	12mo	-0.08	[-0.23 , 0.07]	-0.02	[-0.14 , 0.10]
	24mo	-0.10	[-0.27 , 0.07]	-0.06	[-0.17 , 0.05]
PC Horvath Clock	12mo	-0.08	[-0.14 , -0.02]	-0.07	[-0.12 , -0.02]
	24mo	-0.15	[-0.21 , -0.08]	-0.11	[-0.15 , -0.06]
Hannum Clock	12mo	-0.11	[-0.25 , 0.04]	-0.09	[-0.21 , 0.03]
	24mo	-0.06	[-0.21 , 0.09]	-0.06	[-0.18 , 0.06]
PC Hannum Clock	12mo	-0.07	[-0.15 , 0.02]	-0.07	[-0.14 , 0.01]
	24mo	-0.13	[-0.23 , -0.03]	-0.09	[-0.16 , -0.02]
Skin & Blood Clock	12mo	0.14	[-0.01 , 0.29]	0.03	[-0.08 , 0.14]
	24mo	0.17	[0.01 , 0.33]	0.00	[-0.09 , 0.10]
PC Skin & Blood Clock	12mo	-0.10	[-0.17 , -0.02]	-0.08	[-0.14 , -0.03]
	24mo	-0.19	[-0.26 , -0.11]	-0.12	[-0.17 , -0.07]
**Panel B. Second-Generation Clocks**					
PhenoAge Clock	12mo	0.00	[-0.17 , 0.17]	0.00	[-0.14 , 0.13]
	24mo	-0.14	[-0.34 , 0.06]	0.03	[-0.09 , 0.14]
PC PhenoAge Clock	12mo	-0.02	[-0.15 , 0.11]	-0.06	[-0.17 , 0.04]
	24mo	-0.08	[-0.22 , 0.07]	-0.02	[-0.11 , 0.06]
GrimAge Clock	12mo	-0.07	[-0.18 , 0.04]	-0.19	[-0.28 , -0.09]
	24mo	-0.23	[-0.36 , -0.11]	-0.23	[-0.33 , -0.14]
PC GrimAge Clock	12mo	-0.09	[-0.19 , 0.00]	-0.17	[-0.24 , -0.09]
	24mo	-0.26	[-0.37 , -0.14]	-0.22	[-0.29 , -0.15]
**Panel C. Pace of Aging**					
DunedinPACE	12mo	0.13	[-0.01 , 0.27]	-0.18	[-0.29 , -0.06]
	24mo	0.12	[-0.01 , 0.26]	-0.12	[-0.23 , -0.01]

Table entries for measurements included in primary analysis (PC PhenoAge, PC GrimAge, DunedinPACE) are highlighted in black text.

Table S4. Effect-size estimates for intent-to-treat (ITT) and effect of treatment-on-the-treated (TOT) analysis testing the effect of CALERIE™ intervention on DNA methylation measures of aging. Panel A shows treatment effects for original and PC-based versions[18] of first-generation clocks. Panel B shows treatment effects for original and PC-based versions of second-generation clocks. Panel C shows treatment effects for DunedinPACE. Intent to treat (ITT) effect estimates are reported on the left side of the table. Effect of treatment-on-the-treated (TOT) effect estimates are reported on the right side of the table. ITT analysis was conducted using mixed models to compare change-scores for DNA methylation measures of aging between CR and AL groups. Models included covariates for sex, race/ethnicity, study site, and pre-treatment baseline chronological age and body-mass index stratum. TOT analysis was conducted using instrumental variables regression to test associations of CR dose with aging-measure change-scores. Instruments included treatment group and interactions between treatment group and sex, body-mass index, and baseline levels of the aging measures. Model covariates were the same as in ITT analysis. TOT effect estimates are reported for a 20% dose of CR. This level of CR represents the 75th percentile of the treatment group CR distribution at 12-months follow-up and the 87th percentile of the treatment group CR distribution at 24-months follow-up. Effect-sizes are scaled in terms of standard deviations of the measures at pre-intervention baseline so they can be interpreted on a Cohen's d scale. Effect-sizes can be back-converted into the original units of the measures by multiplying the Cohen's d effect-size by the standard deviation. All tests reported were two-sided. The standard deviations are as follows: Horvath Clock = 3.84; PC Horvath Clock = 4.07; Hannum Clock = 2.85; PC Hannum Clock = 3.50; Skin & Blood Clock = 2.81; PC Skin & Blood Clock = 4.46; PhenoAge Clock = 5.04; PC PhenoAge Clock = 4.84; GrimAge Clock = 2.76; PC GrimAge Clock = 2.82; DunedinPACE = 0.09.

Panel A. First Generation Clocks

		Intent-to-Treat (ITT): Difference between CR and AL			Effect of Treatment on the Treated (TOT): Estimated Effect of 20% CR		
		Treatment Effect (Cohen's d)	95% CI	p-value	Treatment Effect (Cohen's d)	95% CI	p-value
Horvath Clock	12mo	0.07	[-0.11 , 0.25]	0.457	0.14	[-0.10 , 0.39]	0.249
	24mo	0.05	[-0.14 , 0.24]	0.599	0.10	[-0.22 , 0.42]	0.526
PC Horvath Clock	12mo	0.01	[-0.06 , 0.09]	0.771	0.05	[-0.06 , 0.15]	0.375
	24mo	0.04	[-0.04 , 0.12]	0.341	0.05	[-0.08 , 0.19]	0.438
Hannum Clock	12mo	0.03	[-0.15 , 0.21]	0.758	0.09	[-0.15 , 0.33]	0.484
	24mo	0.00	[-0.18 , 0.18]	0.986	-0.02	[-0.35 , 0.31]	0.909
PC Hannum Clock	12mo	0.00	[-0.11 , 0.11]	0.964	0.02	[-0.14 , 0.17]	0.845
	24mo	0.04	[-0.08 , 0.15]	0.501	0.03	[-0.16 , 0.23]	0.753
Skin & Blood Clock	12mo	-0.09	[-0.26 , 0.08]	0.316	-0.06	[-0.30 , 0.18]	0.618
	24mo	-0.14	[-0.32 , 0.04]	0.139	-0.19	[-0.49 , 0.10]	0.197
PC Skin & Blood Clock	12mo	0.02	[-0.07 , 0.11]	0.647	0.05	[-0.07 , 0.17]	0.390
	24mo	0.06	[-0.02 , 0.15]	0.156	0.07	[-0.07 , 0.22]	0.314

Table S4 Continued

Panel B. Second Generation Clocks		Intent-to-Treat (ITT): Difference between CR and AL			Effect of Treatment on the Treated (TOT): Estimated Effect of 20% CR		
		Effect (Cohen's d)	95% CI	p-value	Effect (Cohen's d)	95% CI	p-value
PhenoAge Clock	12mo	0.04	[-0.17 , 0.24]	0.719	-0.02	[-0.29 , 0.26]	0.889
	24mo	0.16	[-0.03 , 0.36]	0.104	0.21	[-0.09 , 0.51]	0.174
PC PhenoAge	12mo	-0.03	[-0.19 , 0.12]	0.663	-0.08	[-0.31 , 0.14]	0.473
	24mo	0.05	[-0.11 , 0.20]	0.550	0.00	[-0.24 , 0.25]	0.977
GrimAge Clock	12mo	-0.10	[-0.23 , 0.04]	0.170	-0.14	[-0.34 , 0.06]	0.164
	24mo	-0.01	[-0.14 , 0.12]	0.898	-0.09	[-0.32 , 0.14]	0.425
PC GrimAge Clock	12mo	-0.04	[-0.16 , 0.07]	0.461	-0.13	[-0.30 , 0.05]	0.154
	24mo	0.05	[-0.07 , 0.17]	0.432	0.01	[-0.17 , 0.19]	0.902
**Panel C. Pace of Aging**							
DunedinPACE	12mo	**-0.29**	**[-0.45 , -0.13]**	**4.83E-04**	**-0.43**	**[-0.67 , -0.19]**	**3.69E-04**
	24mo	**-0.25**	**[-0.41 , -0.09]**	**0.002**	**-0.40**	**[-0.67 , -0.12]**	**0.004**

Table entries for measurements included in primary analysis (PC PhenoAge, PC GrimAge, DunedinPACE) are highlighted in black text. Bolded entries fall below the significance threshold of $p < 0.005$.

Table S5. Analysis of CALERIE™ intervention effects with treatment-group stratified by dose of calorie restriction. Panel A shows treatment effects for original and PC-based versions[18] of first-generation clocks. Panel B shows treatment effects for original and PC-based versions of second-generation clocks. Panel C shows treatment effects for DunedinPACE. The table reports effect-size estimates from analysis using the same regression model used to estimate ITT effects (**Table S4**) with the treatment group stratified by the percent calorie restriction achieved by participants in the CR-intervention group. We used a threshold value of 10% CR relative to baseline to stratify the treatment group. The <10% CR group included n=36 participants at 12-months follow-up and n=48 participants at 24-months follow-up. The >10% CR group included n=89 participants at 12-months follow-up and n=69 participants at 24-months follow-up. All tests reported were two-sided.

Panel A. First-Generation Clocks

			Treatment Effect (Cohen's d)	95% CI	p-value
Horvath Clock	<10% CR	12mo	0.13	[-0.13 , 0.38]	0.331
		24mo	0.07	[-0.15 , 0.29]	0.542
	>10% CR	12mo	0.05	[-0.15 , 0.24]	0.641
		24mo	0.04	[-0.17 , 0.24]	0.740
PC Horvath Clock	<10% CR	12mo	0.00	[-0.10 , 0.11]	0.963
		24mo	0.06	[-0.04 , 0.16]	0.227
	>10% CR	12mo	0.02	[-0.06 , 0.10]	0.713
		24mo	0.02	[-0.06 , 0.10]	0.599
Hannum Clock	<10% CR	12mo	-0.01	[-0.25 , 0.23]	0.945
		24mo	-0.02	[-0.22 , 0.18]	0.857
	>10% CR	12mo	0.04	[-0.15 , 0.24]	0.660
		24mo	0.02	[-0.19 , 0.22]	0.874
PC Hannum Clock	<10% CR	12mo	-0.01	[-0.17 , 0.16]	0.942
		24mo	0.07	[-0.08 , 0.22]	0.347
	>10% CR	12mo	0.01	[-0.11 , 0.12]	0.914
		24mo	0.02	[-0.10 , 0.14]	0.767
Skin & Blood Clock	<10% CR	12mo	-0.09	[-0.30 , 0.13]	0.428
		24mo	-0.18	[-0.39 , 0.03]	0.095
	>10% CR	12mo	-0.09	[-0.28 , 0.10]	0.350
		24mo	-0.11	[-0.31 , 0.09]	0.283
PC Skin & Blood Clock	<10% CR	12mo	0.00	[-0.12 , 0.12]	1.000
		24mo	0.09	[-0.02 , 0.20]	0.105
	>10% CR	12mo	0.03	[-0.06 , 0.12]	0.527
		24mo	0.05	[-0.04 , 0.14]	0.316

Table S5 Continued

Panel B. Second-Generation Clocks

			Treatment Effect (Cohen's d)	95% CI	p-value
PhenoAge Clock	<10% CR	12mo	0.17	[-0.10 , 0.44]	0.219
		24mo	0.10	[-0.13 , 0.34]	0.392
	>10% CR	12mo	-0.02	[-0.24 , 0.21]	0.888
		24mo	0.20	[-0.01 , 0.41]	0.062
PC PhenoAge Clock	<10% CR	12mo	-0.07	[-0.30 , 0.16]	0.540
		24mo	0.05	[-0.13 , 0.24]	0.580
	>10% CR	12mo	-0.02	[-0.19 , 0.14]	0.811
		24mo	0.04	[-0.12 , 0.21]	0.591
GrimAge Clock	<10% CR	12mo	-0.12	[-0.29 , 0.05]	0.167
		24mo	-0.07	[-0.24 , 0.09]	0.383
	>10% CR	12mo	-0.09	[-0.24 , 0.07]	0.266
		24mo	0.04	[-0.10 , 0.18]	0.598
PC GrimAge Clock	<10% CR	12mo	-0.06	[-0.22 , 0.10]	0.492
		24mo	0.05	[-0.09 , 0.19]	0.454
	>10% CR	12mo	-0.04	[-0.17 , 0.09]	0.539
		24mo	0.04	[-0.08 , 0.17]	0.496

Panel C. Pace of Aging

Dunedin PACE	<10% CR	12mo	-0.19	[-0.41 , 0.04]	0.099
		24mo	-0.14	[-0.34 , 0.06]	0.166
	>10% CR	12mo	**-0.33**	**[-0.51 , -0.15]**	**2.31E-04**
		24mo	**-0.33**	**[-0.50 , -0.15]**	**2.63E-04**

Table entries for measurements included in primary analysis (PC PhenoAge, PC GrimAge, DunedinPACE) are highlighted in black text. Bolded entries fall below the significance threshold of $p < 0.005$.

Table S6. Cell-count-adjusted effect-size estimates for intent-to-treat (ITT) and effect of treatment-on-the-treated (TOT) analysis testing the effect of CALERIE™ intervention on DNA methylation measures of aging. Panel A shows treatment effects for original and PC-based versions[18] of first-generation clocks. Panel B shows treatment effects for original and PC-based versions of second-generation clocks. Panel C shows treatment effects for DunedinPACE. Intent to treat (ITT) effect estimates are reported on the left side of the table. Effect of treatment-on-the-treated (TOT) effect estimates are reported on the right side of the table. Models are the same as those reported in **Table S4**, with addition of covariates for change scores of estimated counts of monocytes, lymphocytes (CD4, CD8, natural killer, and b-cells), and neutrophils computed using the R package FlowSorted.Blood.EPIC. All tests reported were two-sided.

		Intent-to-Treat (ITT): Difference between CR and AL			Effect of Treatment on the Treated (TOT): Estimated Effect of 20% CR		
		Treatment Effect (Cohen's d)	95% CI	p-value	Treatment Effect (Cohen's d)	95% CI	p-value
**Panel A. First-Generation Clocks**							
Horvath Clock	12mo	0.11	[-0.07 , 0.29]	0.230	0.16	[-0.09 , 0.42]	0.198
	24mo	0.08	[-0.11 , 0.27]	0.429	0.15	[-0.17 , 0.47]	0.356
PC Horvath Clock	12mo	0.04	[-0.02 , 0.10]	0.197	0.07	[-0.02 , 0.15]	0.123
	24mo	0.03	[-0.03 , 0.09]	0.333	0.08	[-0.03 , 0.19]	0.156
Hannum Clock	12mo	0.06	[-0.12 , 0.23]	0.513	0.09	[-0.15 , 0.34]	0.454
	24mo	-0.01	[-0.18 , 0.17]	0.947	-0.05	[-0.36 , 0.25]	0.725
PC Hannum Clock	12mo	0.06	[-0.01 , 0.14]	0.097	0.09	[-0.02 , 0.19]	0.097
	24mo	0.05	[-0.04 , 0.14]	0.269	0.09	[-0.06 , 0.23]	0.236
Skin & Blood Clock	12mo	-0.09	[-0.26 , 0.09]	0.322	-0.10	[-0.34 , 0.13]	0.384
	24mo	-0.12	[-0.31 , 0.06]	0.187	-0.20	[-0.50 , 0.10]	0.191
PC Skin & Blood Clock	12mo	0.04	[-0.02 , 0.11]	0.206	0.07	[-0.02 , 0.16]	0.111
	24mo	0.04	[-0.03 , 0.11]	0.263	0.08	[-0.03 , 0.19]	0.138
**Panel B. Second-Generation Clocks**							
PhenoAge Clock	12mo	0.04	[-0.14 , 0.22]	0.664	0.05	[-0.21 , 0.30]	0.724
	24mo	0.10	[-0.08 , 0.27]	0.280	0.14	[-0.13 , 0.42]	0.308
PC PhenoAge	12mo	0.01	[-0.06 , 0.08]	0.746	0.02	[-0.08 , 0.11]	0.685
	24mo	0.01	[-0.07 , 0.09]	0.846	0.03	[-0.10 , 0.15]	0.692
GrimAge Clock	12mo	-0.08	[-0.20 , 0.04]	0.213	-0.10	[-0.28 , 0.07]	0.252
	24mo	-0.05	[-0.18 , 0.08]	0.470	-0.10	[-0.32 , 0.13]	0.396
PC GrimAge Clock	12mo	-0.03	[-0.08 , 0.02]	0.248	-0.05	[-0.12 , 0.03]	0.210
	24mo	0.00	[-0.06 , 0.05]	0.884	-0.03	[-0.12 , 0.06]	0.544
**Panel C. Pace of Aging**							
Dunedin PACE	12mo	**-0.26**	**[-0.42 , -0.10]**	**1.29E-03**	**-0.36**	**[-0.59 , -0.13]**	**0.002**
	24mo	**-0.24**	**[-0.40 , -0.07]**	**0.005**	-0.30	[-0.58 , -0.02]	0.033

Table entries for measurements included in primary analysis (PC PhenoAge, PC GrimAge, DunedinPACE) are highlighted in black text. Bolded entries fall below the significance threshold of $p < 0.005$.

Table S7 Means for DNA-methylation measures of aging over follow-up for women and men in the CALERIE™ Trial. Data are the same as reported in **Table S1** disaggregated by sex. Panel A shows values for original and PC-based versions[18] of first-generation clocks. Panel B shows values for original and PC-based versions of second-generation clocks. Panel C shows values for DunedinPACE.

Panel A. First-Generation Clocks

		Women				Men			
		Ad Libitum (n=48)		Calorie Restriction (n=89)		Ad Libitum (n=21)		Calorie Restriction (n=39)	
		Mean	95% CI	Mean	95% CI	Mean	95% CI	Mean	95% CI
Horvath Clock	Baseline	-2.36	[-3.30 , -1.43]	-2.44	[-3.30 , -1.58]	-1.43	[-3.30 , 0.45]	-1.21	[-2.29 , -0.12]
	12mo	-2.39	[-3.37 , -1.41]	-2.45	[-3.34 , -1.57]	-2.54	[-4.68 , -0.40]	-1.22	[-2.29 , -0.15]
	24mo	-2.40	[-3.39 , -1.41]	-2.59	[-3.51 , -1.68]	-2.34	[-4.29 , -0.40]	-1.23	[-2.35 , -0.10]
PC Horvath Clock	Baseline	8.74	[7.62 , 9.87]	8.83	[8.02 , 9.63]	9.77	[7.77 , 11.77]	10.04	[8.68 , 11.40]
	12mo	8.50	[7.25 , 9.75]	8.66	[7.86 , 9.47]	9.20	[7.18 , 11.21]	9.88	[8.57 , 11.19]
	24mo	8.25	[7.04 , 9.45]	8.53	[7.66 , 9.41]	9.33	[7.36 , 11.31]	9.20	[7.76 , 10.63]
Hannum Clock	Baseline	3.54	[2.71 , 4.37]	4.19	[3.61 , 4.76]	4.62	[3.71 , 5.54]	4.13	[3.10 , 5.16]
	12mo	3.41	[2.39 , 4.43]	3.96	[3.32 , 4.59]	3.98	[2.95 , 5.02]	4.02	[3.08 , 4.97]
	24mo	3.54	[2.71 , 4.37]	4.06	[3.40 , 4.71]	4.23	[2.90 , 5.57]	3.80	[2.80 , 4.81]
PC Hannum Clock	Baseline	12.94	[11.95 , 13.93]	13.34	[12.63 , 14.05]	14.57	[12.96 , 16.18]	14.32	[13.21 , 15.44]
	12mo	12.79	[11.64 , 13.94]	13.19	[12.47 , 13.92]	14.27	[12.57 , 15.96]	14.09	[13.05 , 15.14]
	24mo	12.53	[11.49 , 13.57]	13.17	[12.37 , 13.97]	14.41	[12.81 , 16.01]	13.55	[12.43 , 14.68]
Skin & Blood Clock	Baseline	1.60	[0.83 , 2.37]	1.60	[0.99 , 2.21]	0.92	[-0.73 , 2.57]	1.58	[0.94 , 2.23]
	12mo	2.02	[1.21 , 2.83]	1.74	[1.18 , 2.30]	1.06	[-0.32 , 2.45]	1.58	[0.80 , 2.36]
	24mo	2.29	[1.47 , 3.11]	1.70	[1.01 , 2.38]	1.10	[-0.54 , 2.74]	1.66	[1.01 , 2.30]
PC Skin & Blood Clock	Baseline	6.44	[5.10 , 7.78]	6.90	[6.02 , 7.79]	6.43	[4.50 , 8.36]	6.97	[5.47 , 8.46]
	12mo	6.04	[4.55 , 7.53]	6.64	[5.77 , 7.52]	5.90	[3.83 , 7.96]	6.82	[5.38 , 8.25]
	24mo	5.61	[4.19 , 7.04]	6.44	[5.50 , 7.39]	5.92	[3.98 , 7.86]	6.01	[4.44 , 7.58]

Panel B. Second-Generation Clocks

		Women				Men			
		Ad Libitum (n=48)		Calorie Restriction (n=89)		Ad Libitum (n=21)		Calorie Restriction (n=39)	
		Mean	95% CI	Mean	95% CI	Mean	95% CI	Mean	95% CI
PhenoAge Clock	Baseline	-9.56	[-10.88 , -8.23]	-9.50	[-10.58 , -8.43]	-12.28	[-14.34 , -10.22]	-11.59	[-13.15 , -10.04]
	12mo	-10.10	[-11.63 , -8.57]	-9.34	[-10.41 , -8.28]	-11.70	[-13.73 , -9.67]	-11.91	[-13.73 , -10.10]
	24mo	-10.33	[-11.73 , -8.93]	-9.38	[-10.39 , -8.37]	-12.47	[-14.23 , -10.71]	-11.71	[-13.35 , -10.08]
PC PhenoAge Clock	Baseline	2.90	[1.64 , 4.15]	2.96	[1.94 , 3.99]	1.53	[-0.81 , 3.87]	1.98	[0.44 , 3.52]
	12mo	2.83	[1.49 , 4.16]	2.63	[1.67 , 3.58]	1.41	[-0.87 , 3.69]	1.73	[0.06 , 3.41]
	24mo	2.32	[1.02 , 3.61]	3.14	[2.13 , 4.15]	2.05	[-0.15 , 4.25]	1.15	[-0.31 , 2.62]
GrimAge Clock	Baseline	4.65	[3.73 , 5.58]	4.98	[4.42 , 5.54]	5.29	[4.33 , 6.25]	4.31	[3.51 , 5.10]
	12mo	4.44	[3.51 , 5.38]	4.43	[3.87 , 5.00]	5.06	[4.19 , 5.93]	4.07	[3.36 , 4.79]
	24mo	3.97	[3.16 , 4.79]	4.33	[3.72 , 4.94]	4.88	[3.88 , 5.88]	3.50	[2.80 , 4.19]
PC GrimAge Clock	Baseline	12.10	[11.38 , 12.82]	12.81	[12.24 , 13.38]	10.15	[9.08 , 11.21]	9.65	[9.00 , 10.29]
	12mo	11.69	[10.85 , 12.54]	12.35	[11.81 , 12.88]	10.17	[9.00 , 11.35]	9.22	[8.50 , 9.94]
	24mo	11.28	[10.54 , 12.03]	12.11	[11.55 , 12.67]	9.70	[8.61 , 10.79]	8.85	[8.15 , 9.55]

Panel C. Pace of Aging

Dunedin PACE	Baseline	0.94	[0.92 , 0.96]	0.95	[0.93 , 0.96]	0.94	[0.92 , 0.96]	0.95	[0.93 , 0.96]
	12mo	0.95	[0.93 , 0.97]	0.93	[0.92 , 0.94]	0.95	[0.93 , 0.97]	0.93	[0.92 , 0.94]
	24mo	0.95	[0.93 , 0.97]	0.93	[0.92 , 0.95]	0.95	[0.93 , 0.97]	0.93	[0.92 , 0.95]

Table entries for measurements included in primary analysis (PC PhenoAge, PC GrimAge, DunedinPACE) are highlighted in black text.

Table S8. Change scores for DNA-methylation measures of aging at 12-and 24-month follow-ups for women and men in the CALERIE™ Trial. Data are the same as reported in **Table S3** disaggregated by sex. Panel A shows values for original and PC-based versions[18] of first-generation clocks. Panel B shows values for original and PC-based versions of second-generation clocks. Panel C shows values for DunedinPACE.

Panel A. First-Generation Clocks

		Women				Men			
		Ad Libitum (n=48)		Calorie Restriction (n=89)		Ad Libitum (n=21)		Calorie Restriction (n=39)	
		Mean	95% CI	Mean	95% CI	Mean	95% CI	Mean	95% CI
Horvath	12mo	-0.02	[-0.21 , 0.17]	0.00	[-0.15 , 0.16]	-0.21	[-0.46 , 0.04]	-0.07	[-0.26 , 0.12]
Clock	24mo	-0.04	[-0.24 , 0.17]	-0.08	[-0.22 , 0.07]	-0.24	[-0.53 , 0.05]	-0.03	[-0.20 , 0.13]
PC Horvath	12mo	-0.16	[-0.15 , 0.01]	-0.05	[-0.10 , 0.01]	-0.09	[-0.20 , 0.01]	-0.12	[-0.21 , -0.03]
Clock	24mo	0.00	[-0.24 , -0.08]	-0.06	[-0.11 , -0.01]	-0.11	[-0.21 , 0.00]	-0.21	[-0.29 , -0.12]
Hannum	12mo	-0.07	[-0.25 , 0.11]	-0.07	[-0.21 , 0.07]	-0.18	[-0.44 , 0.07]	-0.13	[-0.35 , 0.09]
Clock	24mo	-0.02	[-0.19 , 0.15]	-0.03	[-0.17 , 0.11]	-0.14	[-0.45 , 0.18]	-0.13	[-0.37 , 0.11]
PC Hannum	12mo	-0.06	[-0.17 , 0.05]	-0.04	[-0.12 , 0.05]	-0.07	[-0.22 , 0.07]	-0.14	[-0.27 , 0.00]
Clock	24mo	-0.17	[-0.29 , -0.05]	-0.04	[-0.12 , 0.04]	-0.05	[-0.20 , 0.11]	-0.21	[-0.34 , -0.09]
Skin & Blood	12mo	0.15	[-0.04 , 0.33]	0.06	[-0.07 , 0.19]	0.12	[-0.13 , 0.38]	-0.04	[-0.27 , 0.18]
Clock	24mo	0.22	[0.03 , 0.40]	0.00	[-0.12 , 0.12]	0.06	[-0.27 , 0.39]	0.01	[-0.15 , 0.17]
PC Skin &	12mo	-0.10	[-0.19 , 0.00]	-0.07	[-0.13 , -0.01]	-0.10	[-0.21 , 0.02]	-0.11	[-0.22 , 0.00]
Blood Clock	24mo	-0.22	[-0.32 , -0.12]	-0.09	[-0.15 , -0.03]	-0.11	[-0.21 , -0.01]	-0.21	[-0.29 , -0.12]

Panel B. Second-Generation Clocks

		Women				Men			
		Ad Libitum (n=48)		Calorie Restriction (n=89)		Ad Libitum (n=21)		Calorie Restriction (n=39)	
		Mean	95% CI	Mean	95% CI	Mean	95% CI	Mean	95% CI
PhenoAge	12mo	-0.06	[-0.25 , 0.12]	0.04	[-0.13 , 0.21]	0.14	[-0.23 , 0.50]	-0.11	[-0.31 , 0.08]
Clock	24mo	-0.18	[-0.45 , 0.08]	0.04	[-0.10 , 0.18]	-0.04	[-0.28 , 0.21]	-0.01	[-0.20 , 0.18]
PC PhenoAge	12mo	-0.05	[-0.22 , 0.12]	-0.04	[-0.16 , 0.08]	0.05	[-0.13 , 0.24]	-0.12	[-0.30 , 0.07]
Clock	24mo	-0.16	[-0.34 , 0.03]	0.05	[-0.06 , 0.15]	0.11	[-0.11 , 0.32]	-0.17	[-0.32 , -0.02]
GrimAge	12mo	-0.07	[-0.21 , 0.07]	-0.21	[-0.32 , -0.10]	-0.08	[-0.26 , 0.10]	-0.14	[-0.32 , 0.05]
Clock	24mo	-0.27	[-0.42 , -0.13]	-0.21	[-0.33 , -0.09]	-0.15	[-0.36 , 0.06]	-0.28	[-0.43 , -0.12]
PC GrimAge	12mo	-0.13	[-0.25 , -0.01]	-0.16	[-0.25 , -0.07]	-0.01	[-0.17 , 0.15]	-0.18	[-0.33 , -0.03]
Clock	24mo	-0.30	[-0.44 , -0.15]	-0.18	[-0.27 , -0.10]	-0.16	[-0.32 , 0.00]	-0.29	[-0.41 , -0.17]

Panel C. Pace of Aging

Dunedin	12mo	0.07	[-0.05 , 0.31]	-0.16	[-0.29 , -0.02]	0.14	[-0.08 , 0.36]	-0.22	[-0.46 , 0.02]
PACE	24mo	0.00	[-0.08 , 0.23]	-0.09	[-0.22 , 0.04]	0.23	[-0.04 , 0.51]	-0.20	[-0.40 , 0.01]

Table entries for measurements included in primary analysis (PC PhenoAge, PC GrimAge, DunedinPACE) are highlighted in black text.

Table S9. Effect-estimates from intent-to-treat (ITT) analysis for women and men in the CALERIE™ Trial. The table shows results from ITT analysis stratified by sex and p-values for tests of sex differences in treatment effects. Tests of sex differences were conducted by including a product term testing interaction between treatment group and sex in the ITT models reported in **Table S4**. Panel A shows treatment effects for original and PC-based versions[18] of first-generation clocks. Panel B shows treatment effects for original and PC-based versions of second-generation clocks. Panel C shows treatment effects for DunedinPACE. All tests reported were two-sided.

Panel A. First-Generation Clocks

		Women (AL n=48, CR n=89)			Men (AL n=21, CR n=39)			
		Treatment Effect (Cohen's D)	95% CI	p-value	Treatment Effect (Cohen's D)	95% CI	p-value	p-value for test of sex difference
Horvath Clock	12mo	0.03	[-0.18 , 0.25]	0.763	-0.03	[-0.26 , 0.19]	0.787	0.282
	24mo	0.15	[-0.19 , 0.48]	0.389	0.23	[-0.10 , 0.56]	0.178	0.505
PC Horvath Clock	12mo	0.01	[-0.08 , 0.11]	0.758	0.09	[-0.01 , 0.18]	0.073	0.231
	24mo	0.00	[-0.12 , 0.13]	0.943	-0.07	[-0.20 , 0.06]	0.274	0.082
Hannum Clock	12mo	0.01	[-0.22 , 0.24]	0.934	-0.01	[-0.22 , 0.19]	0.895	0.741
	24mo	0.07	[-0.22 , 0.36]	0.630	0.04	[-0.33 , 0.40]	0.843	0.961
PC Hannum Clock	12mo	0.02	[-0.12 , 0.16]	0.796	0.12	[-0.02 , 0.27]	0.101	0.125
	24mo	-0.03	[-0.20 , 0.14]	0.722	-0.14	[-0.32 , 0.04]	0.127	0.069
Skin & Blood Clock	12mo	-0.09	[-0.30 , 0.13]	0.434	-0.20	[-0.42 , 0.02]	0.068	0.583
	24mo	-0.10	[-0.40 , 0.21]	0.534	0.00	[-0.35 , 0.35]	0.999	0.347
PC Skin & Blood Clock	12mo	0.02	[-0.09 , 0.13]	0.721	0.12	[0.01 , 0.24]	0.037	0.256
	24mo	0.02	[-0.12 , 0.17]	0.747	-0.06	[-0.19 , 0.06]	0.313	0.033

Panel B. Second-Generation Clocks

		Women (AL n=48, CR n=89)			Men (AL n=21, CR n=39)			
		Treatment Effect (Cohen's D)	95% CI	p-value	Treatment Effect (Cohen's D)	95% CI	p-value	p-value for test of sex difference
PhenoAge Clock	12mo	0.13	[-0.11 , 0.37]	0.300	0.21	[-0.05 , 0.46]	0.112	0.201
	24mo	-0.17	[-0.54 , 0.20]	0.361	0.07	[-0.22 , 0.35]	0.640	0.494
PhenoAge Clock	12mo	0.00	[-0.19 , 0.20]	0.996	0.18	[-0.02 , 0.37]	0.072	0.054
	24mo	-0.11	[-0.37 , 0.14]	0.385	-0.24	[-0.48 , 0.01]	0.059	0.083
GrimAge Clock	12mo	-0.12	[-0.28 , 0.05]	0.172	0.06	[-0.10 , 0.22]	0.488	0.585
	24mo	-0.04	[-0.27 , 0.19]	0.723	-0.15	[-0.38 , 0.08]	0.193	0.098
GrimAge Clock	12mo	0.00	[-0.14 , 0.14]	0.988	0.12	[-0.03 , 0.26]	0.117	0.089
	24mo	-0.14	[-0.35 , 0.07]	0.187	-0.10	[-0.30 , 0.09]	0.292	0.558

Panel C. Pace of Aging

Dunedin PACE	12mo	-0.24	[-0.44 , -0.05]	0.015	-0.15	[-0.34 , 0.04]	0.114	0.166
	24mo	-0.39	[-0.69 , -0.09]	0.010	-0.46	[-0.74 , -0.17]	0.002	0.344

Table entries for measurements included in primary analysis (PC PhenoAge, PC GrimAge, DunedinPACE) are highlighted in black text.

Table S10. Effect-estimates from treatment-effect-on-the-treated (TOT) analysis for women and men in the CALERIE™ Trial. The table shows results from TOT analysis stratified by sex and p-values for tests of sex differences in treatment effects. Tests of sex differences were conducted by including a product term testing interaction between treatment group and sex in the TOT models reported in **Table S4**. Panel A shows treatment effects for original and PC-based versions[18] of first-generation clocks. Panel B shows treatment effects for original and PC-based versions of second-generation clocks. Panel C shows treatment effects for DunedinPACE. All tests reported were two-sided.

Panel A. First-Generation Clocks

		Women (AL n=48, CR n=89)			Men (AL n=21, CR n=39)		
		Treatment Effect (Cohen's D)	95% CI	p-value	Treatment Effect (Cohen's D)	95% CI	p-value
Horvath Clock	12mo	0.05	[-0.29 , 0.38]	0.776	0.25	[-0.10 , 0.61]	0.154
	24mo	-0.09	[-0.49 , 0.30]	0.646	0.43	[-0.04 , 0.89]	0.072
PC Horvath Clock	12mo	0.09	[-0.07 , 0.24]	0.267	0.03	[-0.10 , 0.16]	0.604
	24mo	0.16	[-0.03 , 0.34]	0.099	-0.08	[-0.31 , 0.14]	0.463
Hannum Clock	12mo	0.07	[-0.29 , 0.42]	0.707	0.15	[-0.21 , 0.50]	0.417
	24mo	0.05	[-0.36 , 0.45]	0.823	-0.01	[-0.57 , 0.56]	0.979
PC Hannum Clock	12mo	0.08	[-0.15 , 0.30]	0.512	-0.05	[-0.25 , 0.15]	0.600
	24mo	0.20	[-0.07 , 0.48]	0.151	-0.22	[-0.52 , 0.07]	0.131
Skin & Blood Clock	12mo	-0.06	[-0.41 , 0.29]	0.742	0.01	[-0.37 , 0.38]	0.963
	24mo	-0.33	[-0.78 , 0.12]	0.151	-0.12	[-0.63 , 0.39]	0.651
PC Skin & Blood Clock	12mo	0.08	[-0.09 , 0.26]	0.349	0.03	[-0.15 , 0.20]	0.774
	24mo	0.17	[-0.04 , 0.38]	0.113	-0.09	[-0.29 , 0.12]	0.396

Panel B. Second-Generation Clocks

		Women (AL n=48, CR n=89)			Men (AL n=21, CR n=39)		
		Treatment Effect (Cohen's D)	95% CI	p-value	Treatment Effect (Cohen's D)	95% CI	p-value
PhenoAge Clock	12mo	0.16	[-0.20 , 0.53]	0.385	-0.26	[-0.63 , 0.11]	0.165
	24mo	0.31	[-0.15 , 0.76]	0.185	0.07	[-0.38 , 0.52]	0.765
PC PhenoAge Clock	12mo	0.01	[-0.32 , 0.34]	0.966	-0.17	[-0.49 , 0.15]	0.303
	24mo	0.30	[-0.05 , 0.65]	0.092	-0.40	[-0.76 , -0.04]	0.028
GrimAge Clock	12mo	-0.27	[-0.55 , 0.01]	0.056	-0.08	[-0.36 , 0.21]	0.597
	24mo	0.01	[-0.32 , 0.34]	0.952	-0.45	[-0.81 , -0.08]	0.017
PC GrimAge Clock	12mo	-0.02	[-0.25 , 0.21]	0.855	-0.25	[-0.51 , 0.01]	0.061
	24mo	0.22	[-0.03 , 0.47]	0.091	-0.30	[-0.58 , -0.03]	0.030

Panel C. Pace of Aging

DunedinPACE	**12mo**	**-0.42**	**[-0.73 , -0.11]**	**0.007**	**-0.59**	**[-0.93 , -0.25]**	**0.001**
	24mo	-0.32	[-0.69 , 0.04]	0.081	**-0.76**	**[-1.21 , -0.30]**	**0.001**

Table entries for measurements included in primary analysis (PC PhenoAge, PC GrimAge, DunedinPACE) are highlighted in black text. Bolded entries fall below the significance threshold of $p < 0.005$.

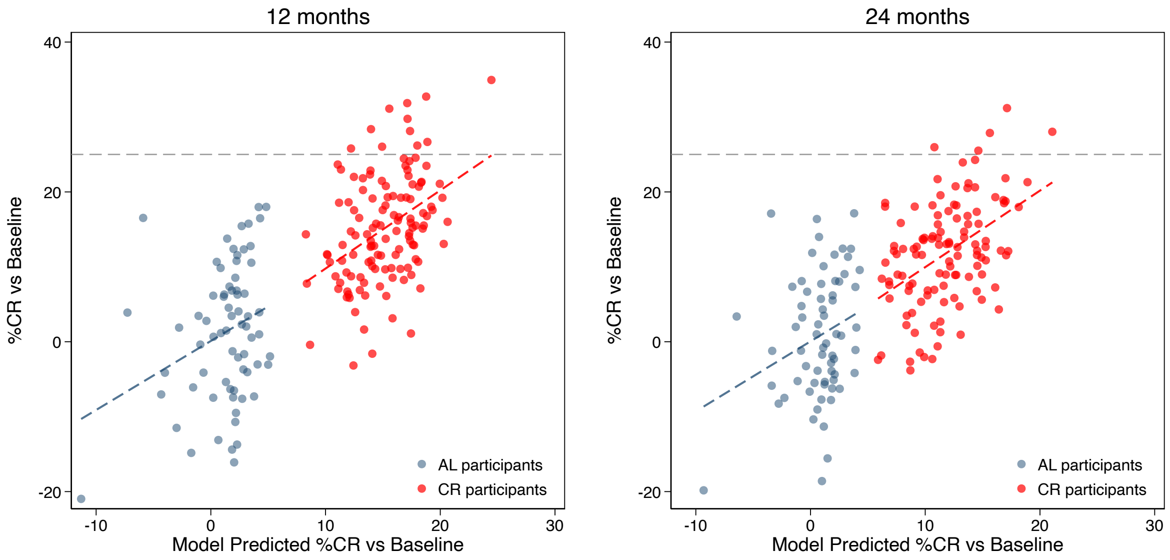

Figure S1. Actual %CR plotted against Predicted CR. The figure plots the measured % CR vs baseline (y-axis) against the model-predicted CR (x-axis) at the 12- (left) and 24-month (right) follow-up assessments. Values for ad-libitum (AL) control-group participants are plotted in blue. Values for caloric restriction (CR) intervention-group participants are plotted in red. The intervention target of 25% CR is noted with a dashed gray line intersecting the y-axis. Predicted values are derived from the base model for the first stage of IV regression analysis described in equation (1). The model includes terms for intervention condition, sex, pre-treatment baseline body-mass index, interactions among these variables, and covariates for pre-treatment baseline chronological age, study site, and race/ethnicity.

Figure S2. Associations of DNA methylation measures of aging with chronological age at pre-intervention baseline in the CALERIE™ Trial. Figures show scatter plots of the DNA methylation measures of aging (Y-axis) against chronological age (X-axis) for women (blue circles) and men (purple triangles) and fitted regression slopes. Correlations with chronological age are as follows: Horvath Clock r=0.87, PC Horvath Clock r=0.84, Hannum Clock r=0.92, PC Hannum Clock r=0.88, Skin & Blood Clock r=0.94, PC Skin & Blood Clock r=0.80, PhenoAge Clock r=0.84, PC PhenoAge Clock r=0.85, GrimAge Clock r=0.93, PC GrimAge Clock r=0.92, DunedinPACE r=0.15.

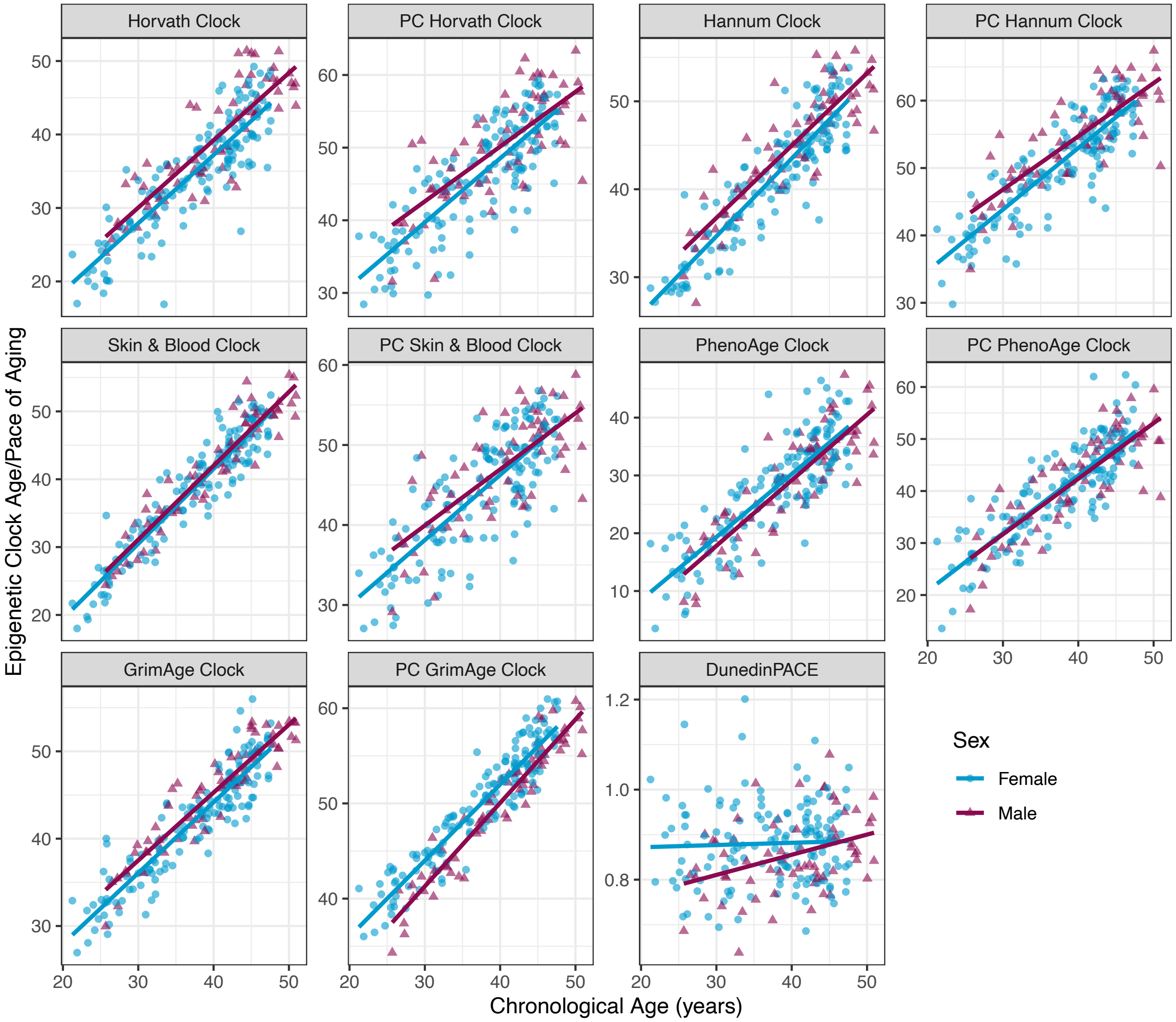

Figure S3. Change from baseline to 12- and 24-month follow-up in DNA methylation measures of aging in the ad libitum (AL) and caloric restriction (CR) groups in the CALERIE™ Trial. Values for the AL control group (n = 69 participants) are graphed in blue. Values for the CR treatment group (n = 128 participants) are graphed in red. Panel A mean values of change from baseline and 95% confidence intervals estimated from mixed models at the 12- and 24-month follow-ups for the AL and CR groups. There is no confidence interval estimated for baseline because change from baseline is exactly zero at this timepoint. Panel B shows box plots of the observed values of the measures at baseline and 12- and 24-month follow-ups. The boxes show the interquartile range; the whiskers show 1.5x the interquartile range; the center line shows the median; individual participant data are plotted as dots and connected with lines. Change scores are denominated in "years" of DNA methylation age. For the clocks, expected change under the null hypothesis is 1 year at 12-months follow-up and 2 years at 24 months follow-up.

Panel A

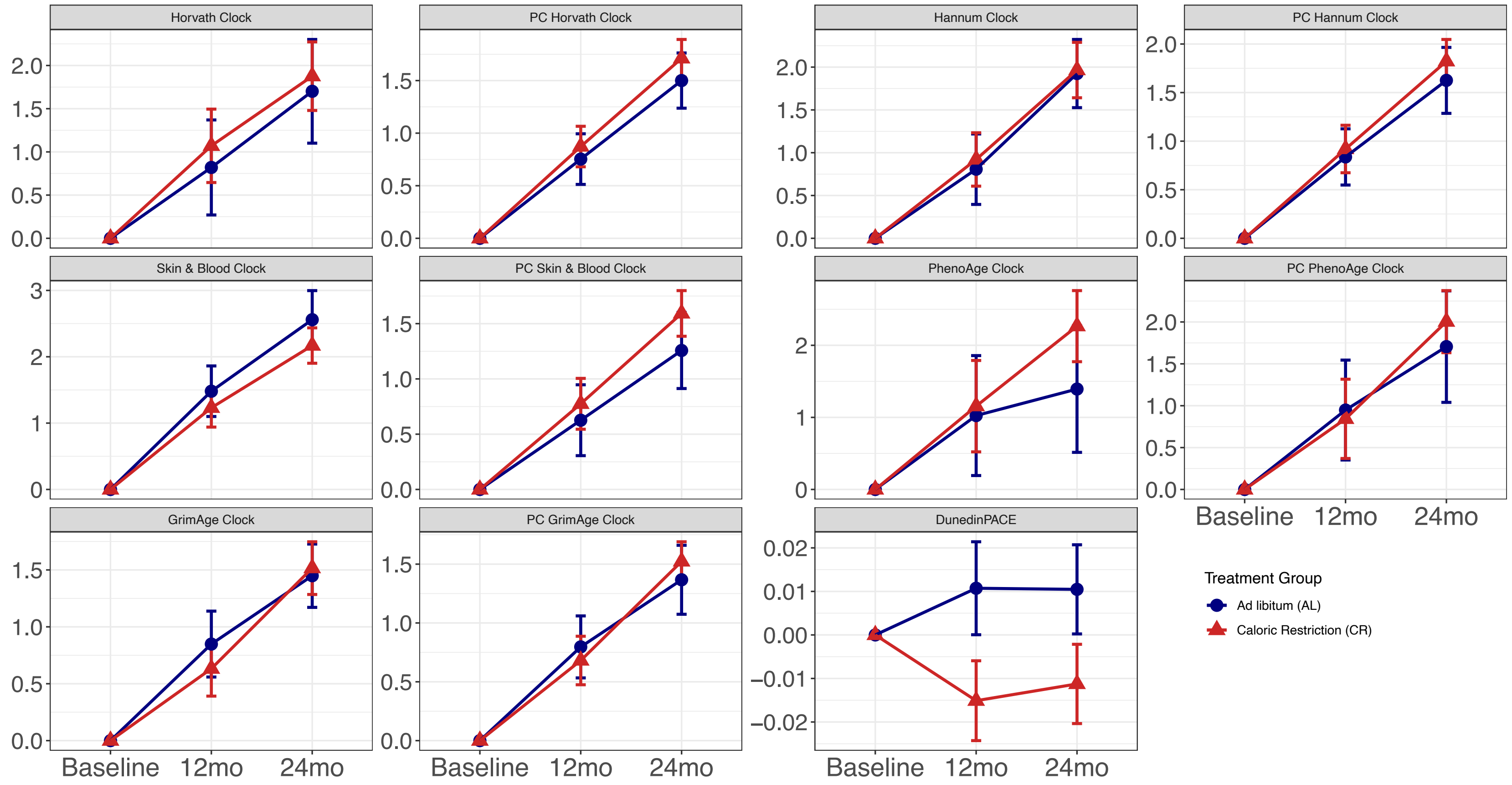
Horvath Clock
PC Horvath Clock
Hannum Clock
PC Hannum Clock
Skin & Blood Clock
PC Skin & Blood Clock
PhenoAge Clock
PC PhenoAge Clock
GrimAge Clock
PC GrimAge Clock
DunedinPACE
Baseline
12mo
24mo
Treatment Group
Ad libitum (AL)
Caloric Restriction (CR)

Figure S3 Continued

Panel B

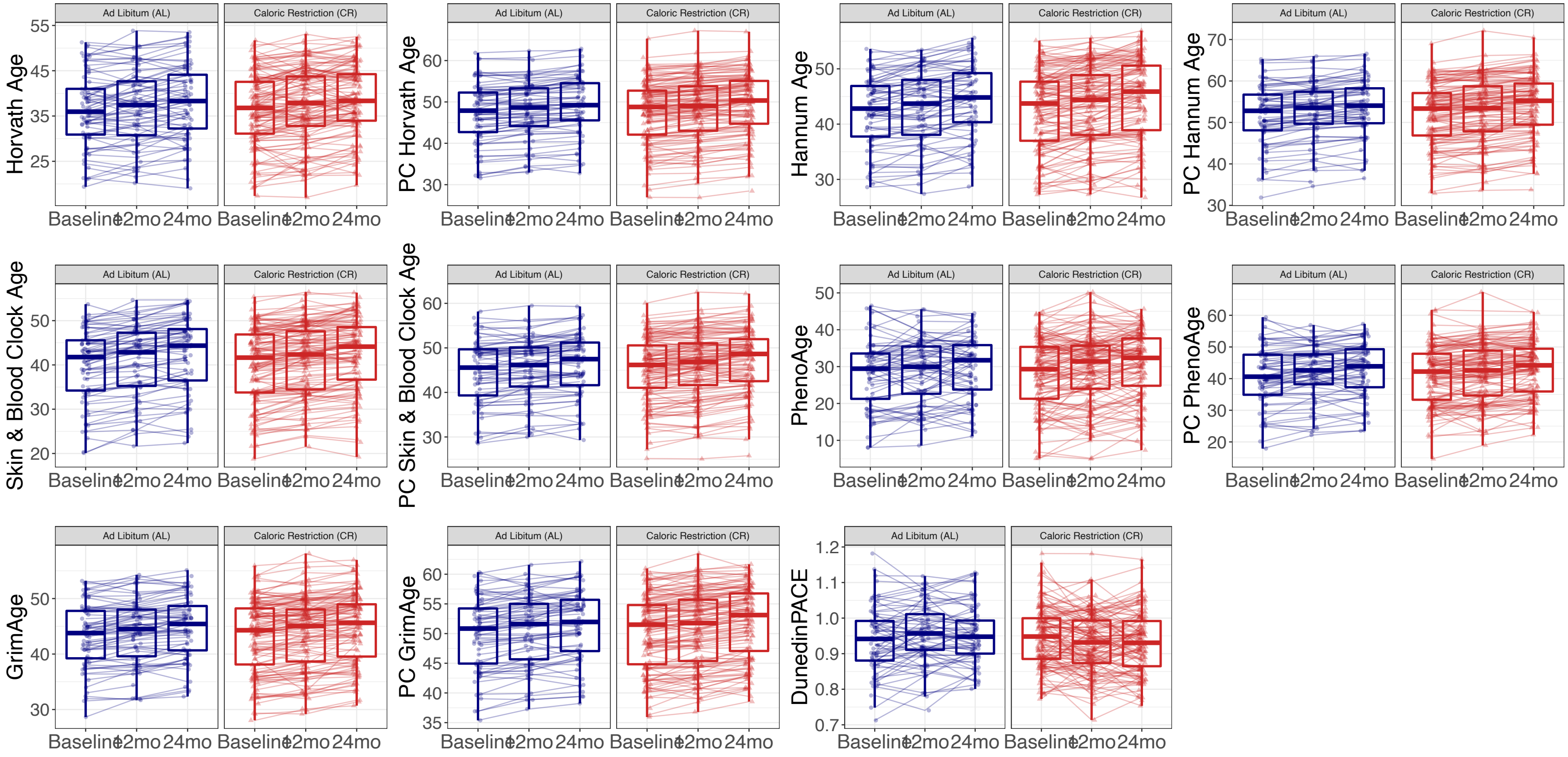

Figure S4. Association of DunedinPACE with clinical measures modified by CALERIE™ intervention. To evaluate whether intervention-induced changes in DunedinPACE overlapped with intervention effects on clinical measures, we conducted analysis of clinical measures altered by CALERIE™ intervention as reported in prior publications by our group[2,28]. Panel A shows effect-sizes (and 95% confidence intervals) for associations between DunedinPACE and each of the clinical measures at pre-intervention baseline (n = 197). Effect-sizes are graphed as Pearson r to be comparable across clinical measures with different scales. Panel B graphs estimates of total effects of CALERIE™ treatment on each of the clinical measures (blue bars) and indirect effects mediated through DunedinPACE (red bars). Effect estimates were generated according to the equations published by Imai et al.[29,29] implemented with the Stata package 'medeff'. Treatment effects are reported on a standard deviation scale (Cohen's d) so they are comparable between clinical measures. Confidence intervals did not overlap zero for estimates of mediation percent for log CRP, HDL, Triglycerides, log Triglycerides, Insulin AUC, SBP, DBP, Waist Circumference, Metabolic Syndrome, KDM BA, PhenoAge. For estimates of the adjusted causal mediation effect (ACME), confidence intervals overlapped zero for all outcomes with the exception of log-CRP and PhenoAge.

Panel A.

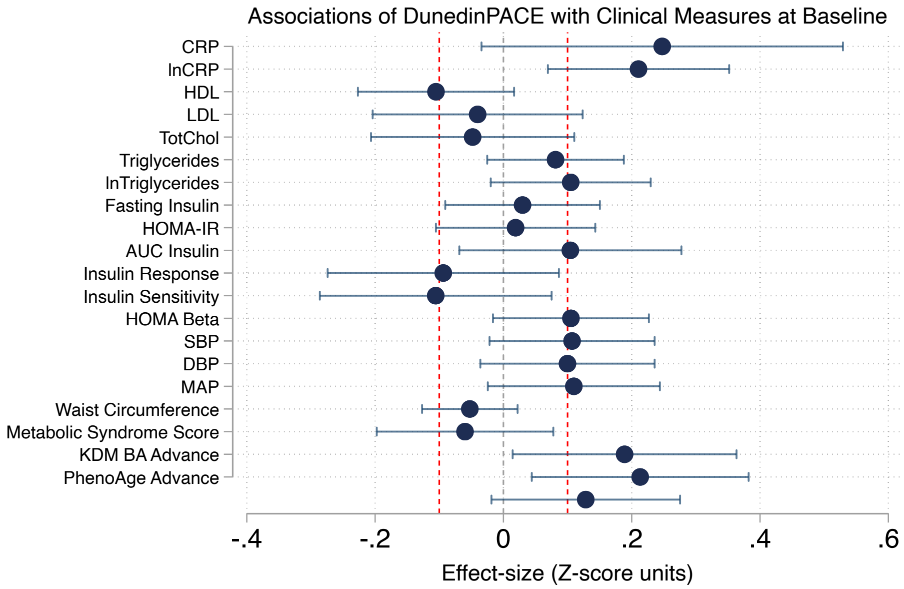

Figure S4 Continued

Panel B.

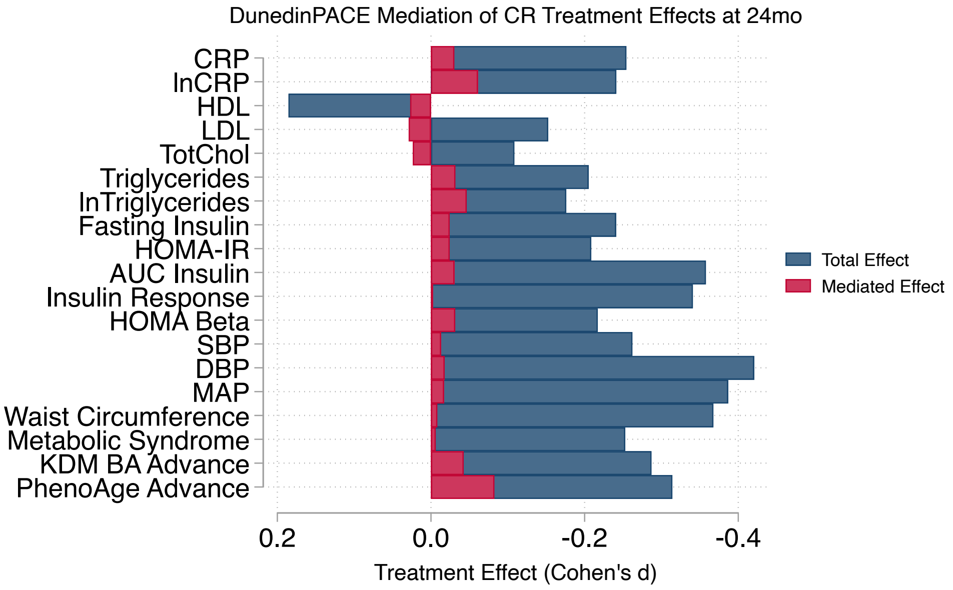

REFERENCES

1. Ravussin, E. *et al.* A 2-Year Randomized Controlled Trial of Human Caloric Restriction: Feasibility and Effects on Predictors of Health Span and Longevity. *J. Gerontol. A. Biol. Sci. Med. Sci.* **70**, 1097–1104 (2015).
2. Kraus, W. E. *et al.* 2 years of calorie restriction and cardiometabolic risk (CALERIE): exploratory outcomes of a multicentre, phase 2, randomised controlled trial. *Lancet Diabetes Endocrinol.* **0**, (2019).
3. Racette, S. B. *et al.* Approaches for quantifying energy intake and %calorie restriction during calorie restriction interventions in humans: the multicenter CALERIE study. *Am. J. Physiol. - Endocrinol. Metab.* **302**, E441–E448 (2012).
4. Das, S. K. *et al.* Body-composition changes in the Comprehensive Assessment of Long-term Effects of Reducing Intake of Energy (CALERIE)-2 study: a 2-y randomized controlled trial of calorie restriction in nonobese humans. *Am. J. Clin. Nutr.* **105**, 913–927 (2017).
5. Davis, S. *et al.* methylumi: Handle Illumina methylation data. (2021) doi:10.18129/B9.bioc.methylumi.
6. Huber, W. *et al.* Orchestrating high-throughput genomic analysis with Bioconductor. *Nat. Methods* **12**, 115–121 (2015).
7. Lehne, B. *et al.* A coherent approach for analysis of the Illumina HumanMethylation450 BeadChip improves data quality and performance in epigenome-wide association studies. *Genome Biol.* **16**, 37 (2015).
8. Salas, L. A. *et al.* An optimized library for reference-based deconvolution of whole-blood biospecimens assayed using the Illumina HumanMethylationEPIC BeadArray. *Genome Biol.* **19**, 64 (2018).

9. Aryee, M. J. *et al.* Minfi: a flexible and comprehensive Bioconductor package for the analysis of Infinium DNA methylation microarrays. *Bioinformatics* **30**, 1363–1369 (2014).
10. Horvath, S. & Raj, K. DNA methylation-based biomarkers and the epigenetic clock theory of ageing. *Nat. Rev. Genet.* **19**, 371–384 (2018).
11. Horvath, S. DNA methylation age of human tissues and cell types. *Genome Biol.* **14**, R115 (2013).
12. Hannum, G. *et al.* Genome-wide methylation profiles reveal quantitative views of human aging rates. *Mol. Cell* **49**, 359–367 (2013).
13. Chen, B. H. *et al.* DNA methylation-based measures of biological age: meta-analysis predicting time to death. *Aging* **8**, 1844–1865 (2016).
14. Lu, A. T. *et al.* DNA methylation GrimAge strongly predicts lifespan and healthspan. *Aging* **11**, 303–327 (2019).
15. Levine, M. E. *et al.* An epigenetic biomarker of aging for lifespan and healthspan. *Aging* **10**, 573–591 (2018).
16. Levine, M. E. Assessment of Epigenetic Clocks as Biomarkers of Aging in Basic and Population Research. *J. Gerontol. Ser. A* **75**, 463–465 (2020).
17. Fahy, G. M. *et al.* Reversal of epigenetic aging and immunosenescent trends in humans. *Aging Cell* **18**, e13028 (2019).
18. Higgins-Chen, A. T. *et al.* A computational solution for bolstering reliability of epigenetic clocks: implications for clinical trials and longitudinal tracking. *Nat. Aging* **2**, 644–661 (2022).
19. Belsky, D. W. *et al.* DunedinPACE, a DNA methylation biomarker of the pace of aging. *eLife* **11**, e73420 (2022).

20. Belsky, D. W. *et al.* Quantification of biological aging in young adults. *Proc. Natl. Acad. Sci. U. S. A.* **112**, E4104-4110 (2015).
21. Elliott, M. L. *et al.* Disparities in the pace of biological aging among midlife adults of the same chronological age have implications for future frailty risk and policy. *Nat. Aging* **1**, 295–308 (2021).
22. Sugden, K. *et al.* Patterns of Reliability: Assessing the Reproducibility and Integrity of DNA Methylation Measurement. *Patterns* **1**, 100014 (2020).
23. StataCorp, L. Stata multilevel mixed-effects reference manual. *Coll. Stn. TX StataCorp LP* **9**, (2013).
24. Sussman, J. B. & Hayward, R. A. An IV for the RCT: using instrumental variables to adjust for treatment contamination in randomised controlled trials. *BMJ* **340**, c2073 (2010).
25. Baum, C. F., Schaffer, M. E. & Stillman, S. Instrumental variables and GMM: Estimation and testing. *Stata J.* **3**, 1–31 (2003).
26. Bang, H. & Davis, C. E. On estimating treatment effects under non-compliance in randomized clinical trials: are intent-to-treat or instrumental variables analyses perfect solutions? *Stat. Med.* **26**, 954–964 (2007).
27. McEwen, L. M. *et al.* Systematic evaluation of DNA methylation age estimation with common preprocessing methods and the Infinium MethylationEPIC BeadChip array. *Clin. Epigenetics* **10**, 123 (2018).
28. Kwon, D. & Belsky, D. W. A toolkit for quantification of biological age from blood chemistry and organ function test data: BioAge. *GeroScience* **43**, 2795–2808 (2021).
29. Imai, K., Keele, L. & Yamamoto, T. Identification, inference and sensitivity analysis for causal mediation effects. *Stat. Sci.* **25**, 51–71 (2010).
