# Supplementary material for: Effect of long-term caloric restriction on DNA methylation measures of biological aging in healthy adults from the CALERIE trial
Source: Nat Aging. 2023 Feb 9;3(3):248–57. doi: 10.1038/s43587-022-00357-y (PMC10148951; doi:10.1038/s43587-022-00357-y)
Supplement: Supplementary file 2 — Reporting Summary [file 43587_2022_357_MOESM2_ESM.pdf]

nature portfolio

Corresponding author(s): Daniel W Belsky

Last updated by author(s): 12-10-2021

Reporting Summary

Nature Portfolio wishes to improve the reproducibility of the work that we publish. This form provides structure for consistency and transparency in reporting. For further information on Nature Portfolio policies, see our Editorial Policies and the Editorial Policy Checklist.

Statistics

For all statistical analyses, confirm that the following items are present in the figure legend, table legend, main text, or Methods section.

n/a	Confirmed	
☐	☒	The exact sample size (*n*) for each experimental group/condition, given as a discrete number and unit of measurement
☐	☒	A statement on whether measurements were taken from distinct samples or whether the same sample was measured repeatedly
☐	☒	The statistical test(s) used AND whether they are one- or two-sided<br>*Only common tests should be described solely by name; describe more complex techniques in the Methods section.*
☐	☒	A description of all covariates tested
☐	☒	A description of any assumptions or corrections, such as tests of normality and adjustment for multiple comparisons
☐	☒	A full description of the statistical parameters including central tendency (e.g. means) or other basic estimates (e.g. regression coefficient) AND variation (e.g. standard deviation) or associated estimates of uncertainty (e.g. confidence intervals)
☐	☒	For null hypothesis testing, the test statistic (e.g. *F*, *t*, *r*) with confidence intervals, effect sizes, degrees of freedom and *P* value noted<br>*Give P values as exact values whenever suitable.*
☒	☐	For Bayesian analysis, information on the choice of priors and Markov chain Monte Carlo settings
☐	☒	For hierarchical and complex designs, identification of the appropriate level for tests and full reporting of outcomes
☐	☒	Estimates of effect sizes (e.g. Cohen's *d*, Pearson's *r*), indicating how they were calculated

Our web collection on statistics for biologists contains articles on many of the points above.

Software and code

Policy information about availability of computer code

Data collection	DNA methylation data: Quality control and normalization analyses were performed using the methylumi (v. 2.32.0) 30 Bioconductor (v 2.46.0) 31 package for the R statistical programming environment (v 3.6.3).
Data analysis	Cell count estimation was performed using the Houseman Equation via the minfi (1.44.0) and FlowSorted.Blood.EPIC (2.2.0) R packages. DNA methylation measures of aging were computed using the software hosted by the Horvath Lab (epigenetic clocks) and R code publicly availabel on GitHub (Pace of Aging Measures; https://github.com/danbelsky). Analyses were conducted using the Stata Software (version 15.0). Stata code used to conduct analysis will be made available on GitHub upon acceptance of the article for publication.

For manuscripts utilizing custom algorithms or software that are central to the research but not yet described in published literature, software must be made available to editors and reviewers. We strongly encourage code deposition in a community repository (e.g. GitHub). See the Nature Portfolio guidelines for submitting code & software for further information.

Data

Policy information about availability of data

All manuscripts must include a data availability statement. This statement should provide the following information, where applicable:

- Accession codes, unique identifiers, or web links for publicly available datasets
- A description of any restrictions on data availability
- For clinical datasets or third party data, please ensure that the statement adheres to our policy

All non-genomic data are available from the CALERIE biorepository (calerie.duke.edu). Genomic data are available from corresponding author until March 2024, at which point they will be available from the CALERIE biorepository.

Field-specific reporting

Please select the one below that is the best fit for your research. If you are not sure, read the appropriate sections before making your selection.

☒ Life sciences ☐ Behavioural & social sciences ☐ Ecological, evolutionary & environmental sciences

For a reference copy of the document with all sections, see nature.com/documents/nr-reporting-summary-flat.pdf

Life sciences study design

All studies must disclose on these points even when the disclosure is negative.

Sample size	N=197
Data exclusions	Data for n=23 participants were excluded due to missing or low quality DNA methylation data.
Replication	CALERIE is the only trial of long-term CR in healthy, non-obese humans. No replication was undertaken.
Randomization	After baseline testing, participants were randomly assigned at a ratio of 2:1 to a CR behavioral intervention or to an ad libitum control group (AL). Randomization was stratified by site, sex, and BMI. A permuted block randomization technique was used.
Blinding	All study personnel doing assessments were masked to treatment assignment.

Reporting for specific materials, systems and methods

We require information from authors about some types of materials, experimental systems and methods used in many studies. Here, indicate whether each material, system or method listed is relevant to your study. If you are not sure if a list item applies to your research, read the appropriate section before selecting a response.

Materials & experimental systems

n/a	Involved in the study	
☒	☐	Antibodies
☒	☐	Eukaryotic cell lines
☒	☐	Palaeontology and archaeology
☒	☐	Animals and other organisms
☐	☒	Human research participants
☐	☒	Clinical data
☒	☐	Dual use research of concern

Methods

n/a	Involved in the study	
☒	☐	ChIP-seq
☒	☐	Flow cytometry
☒	☐	MRI-based neuroimaging

Human research participants

Policy information about studies involving human research participants

Population characteristics	Of 238 eligible individuals, CALERIE randomized N=220 participants to treatment (145 CR-intervention and 75 AL-control; Figure 1). Blood DNAm data were generated at baseline and at least one follow-up timepoint for n=197 participants (128 CR and 69 AL). Of this analysis sample, n=105 (82%) of CR participants and n=59 (86%) of AL participants had DNAm data available from all three time points (baseline, 12-months, and 24-months). Participants had mean age of 38 years (SD=7), 70% were women, and 77% were white; there were no differences in age, sex, race/ethnicity between AL and CR at baseline (Table 1).
Recruitment	Our study involved analysis of stored biospecimens and analysis of already-collected de-identified secondary data. Briefly, the details of the parent study's recruitment are as follows: CALERIE's age range (21–50 years) was selected to be comparable to the life stage when many "adult onset" CR studies showing sub- stantial effects on life span and aging were begun in rodents. The body mass index (BMI) range (22.0 ≤ BMI < 28 kg/m2) was selected to examine CR's effects in both normal weight and moderately over- weight persons. Exclusion criteria (detailed in Rochon J, Bales CW, Ravussin E, et al.; CALERIE Study Group. Design and conduct of the CALERIE study: comprehensive assessment of the long-term effects of reducing intake of energy. J Gerontol A Biol Sci Med Sci. 2011;66:97–108. doi:10.1093/gerona/glq168) included significant medical conditions (eg, cardiovascular disease or diabetes), abnormal laboratory markers (eg, elevated potassium, or below-normal hemoglobin levels), present or potential psychiatric or behavioral problems (eg, eating disorders or depressive symptoms), regular use of medications except oral contraceptives, current smoking, a high level of regular physical activity, and pregnancy. See Stewart TM, Bhapkar M, Das S, et al.; CALERIE Study Group. Comprehensive Assessment of Long-term Effects of Reducing Intake of Energy Phase 2 (CALERIE Phase 2) screening and recruitment: methods and results. Con- temp Clin Trials. 2013;34:10–20. doi:10.1016/j.cct.2012.08.011 for more details.

Ethics oversight	Oversight of our study was performed by the Institutional Review Board of Columbia University Irving Medical Center AAAS2948.

Note that full information on the approval of the study protocol must also be provided in the manuscript.

Clinical data

Policy information about clinical studies

All manuscripts should comply with the ICMJE guidelines for publication of clinical research and a completed CONSORT checklist must be included with all submissions.

Clinical trial registration	NCT00427193
Study protocol	https://calerie.duke.edu/protocols-procedures
Data collection	CALERIE™ Phase-2, was a multi-center, randomized controlled trial conducted at three clinical centers in the USA10 (ClinicalTrials.gov Identifier: NCT00427193). It aimed to evaluate the time-course effects of 25% CR (i.e. intake 25% below the individual's baseline level) over a 2-year period in healthy, adults (men aged 21–50 y, premenopausal women aged 21–47 y) with BMI in the normal weight or slightly overweight range (BMI 22.0-27.9 kg/m2). The study protocol was approved by Institutional Review Boards at three clinical centers (Washington University School of Medicine, St Louis, MO, USA; Pennington Biomedical Research Center, Baton Rouge, LA, USA; Tufts University, Boston, MA, USA) and the coordinating center at Duke University (Durham, NC, USA). All study participants provided written informed consent.
Outcomes	The primary outcomes were three DNA methylation measures of aging (PC PhenoAge, PC GrimAge, and DunedinPACE). We selected these based on published evidence of measurement reliability and criterion validity (i.e. associations with aging-related morbidity and mortality). Secondary outcomes were the original versions of the PhenoAge and GrimAge DNA methylation clocks, and the original and PC versions of DNA methylation clocks proposed by Horvath (multi-tissue and Skin & Blood) and Hannum et al.
